# Novel in vivo models of autosomal optic atrophy reveal conserved pathological changes in neuronal mitochondrial structure and function

**DOI:** 10.1096/fj.202403271R

**Published:** 2025-04-09

**Authors:** Elin L. Strachan, Eugene T. Dillon, Mairéad Sullivan, Jeffrey C. Glennon, Amandine Peyrel, Jérôme Sarniguet, Kevin Dubois, Benjamin Delprat, Breandán N. Kennedy, Niamh C. O'Sullivan

**Affiliations:** ^1^ UCD Conway Institute of Biomolecular and Biomedical Research University College Dublin Dublin Ireland; ^2^ UCD School of Biomolecular and Biomedical Science University College Dublin Dublin Ireland; ^3^ UCD School of Medicine University College Dublin Dublin Ireland; ^4^ MMDN University of Montpellier, EPHE, INSERM Montpellier France

**Keywords:** *Drosophila*, mitochondria, optic atrophy, visual impairment, zebrafish

## Abstract

Autosomal optic atrophy (AOA) is a form of hereditary optic neuropathy characterized by the irreversible and progressive degermation of the retinal ganglion cells. Most cases of AOA are associated with a single dominant mutation in *OPA1*, which encodes a protein required for fusion of the inner mitochondrial membrane. It is unclear how loss of OPA1 leads to neuronal death, and despite ubiquitous expression appears to disproportionately affect the RGCs. This study introduces two novel in vivo models of OPA1‐mediated AOA, including the first developmentally viable vertebrate *Opa1* knockout (KO). These models allow for the study of *Opa1* loss in neurons, specifically RGCs. Though survival is significantly reduced in *Opa1* deficient zebrafish and *Drosophila*, both models permit the study of viable larvae. Moreover, zebrafish *Opa1* KO larvae show impaired visual function but unchanged locomotor function, indicating that retinal neurons are particularly sensitive to *Opa1* loss. Proteomic profiling of both models reveals marked disruption in protein expression associated with mitochondrial function, consistent with an observed decrease in mitochondrial respiratory function. Similarly, mitochondrial fragmentation and disordered cristae organization were observed in neuronal axons in both models highlighting *Opa1*'s highly conserved role in regulating mitochondrial morphology and function in neuronal axons. Importantly, in *Opa1* deficient zebrafish, mitochondrial disruption and visual impairment precede degeneration of RGCs. These novel models mimic key features of AOA and provide valuable tools for therapeutic screening. Our findings suggest that therapies enhancing mitochondrial function may offer a potential treatment strategy for AOA.

## Graphical abstract

Autosomal optic atrophy (AOA) is a hereditary condition caused by mutations in *OPA1*, leading to retinal ganglion cell (RGC) degeneration. This study introduces two novel models to study *OPA1* loss in neurons, revealing reduced life span and impaired visual function. Moreover, we find that neuronal mitochondrial dysfunction precedes RGC degeneration. These models offer tools for potential therapeutic development.

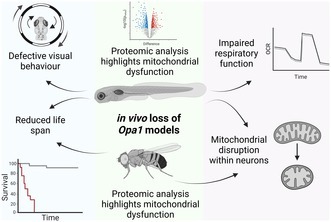

## 1 INTRODUCTION

Autosomal optic atrophy (AOA) is a form of inherited retinal disease affecting around 1 in 30 000 individuals, although it can have an incidence as high as 1 in 12 000 in some populations.^1^, ^2^ AOA is characterized by the progressive and irreversible degeneration of the retinal ganglion cells (RGCs), accompanied by the loss of vision—often with color vision deficits and centrocecal, central, or paracentral scotoma.^3^, ^4^ The extent of visual impairment is also variable among affected individuals, ranging from visual blindness to relatively mild deficits.^4^, ^5^ Although vision loss is usually slow, there is some variability among patients, and more rapid decline has been observed.^4^, ^6^ A subset of AOA individuals—approximately 20%—additionally experience extra‐ocular symptoms (AOA+), including sensorineural deafness, ataxia, peripheral neuropathy, parkinsonism, and myopathy.^7^ The most common cause of AOA is mutations in the ubiquitously expressed, highly conserved gene optic atrophy 1 (*OPA1*),^2^, ^8^ a mitochondrially localized dynamin‐like protein. There are over 900 known or likely pathogenic mutations of *OPA1*, located throughout the gene; however, the dynamin and GTPase domains appear overrepresented (Refs. [^7^, ^9^]; https://databases.lovd.nl/shared/variants/OPA1/unique). Most pathogenic *OPA1* mutations appear to result from haploinsufficiency, evidenced by a reduction in OPA1 protein or mRNA, suggesting that diminished OPA1 is the underlying cause of disease.^10^, ^11^ Missense mutations are often associated with AOA+ and more severe ocular symptoms.^4^, ^7^, ^12^

*OPA1* encodes a dynamin‐like GTPase localized to the inner mitochondrial membrane (IMM) which is essential for IMM fusion following the fusion of the outer mitochondrial membrane (OMM) by the mitofusins (MFN1 and MFN2).^13^ OPA1 exists within the cell as 2 groups of isoforms, short and long (s‐OPA1 and l‐OPA1 respectively). The l‐form possesses a transmembrane region on the N‐terminus and undergoes cleavage by the proteases YME1 Like 1 ATPase (YME1L) and OMA1 Zinc Metallopeptidase (OMA1) to produce the soluble short form.^14^, ^15^ OPA1 dimerises, promoting the formation of helical assemblies causing the IMM to bud and produce curvature stress which, along with bringing the opposing IMMs into close proximity, is able to drive fusion.^16^, ^17^ OPA1 is also important for the morphogenesis, organization, and maintenance of mitochondrial cristae, usually in collaboration with components of the MICOS (mitochondria contact site and cristae organizing system) complex. Specifically, OPA1 appears to control the width, stability, and shape of cristae junctions and lumen in collaboration with MICOS proteins such as MIC60.^18^ *OPA1* deficient cells exhibit highly fragmented mitochondria, exhibiting a reduced number of cristae with highly abnormal morphology.^19^ The presence of both the s‐OPA1 and l‐OPA1 isoforms is required to regulate IMM fusion and to maintain normal cristae morphology.^15^, ^20^, ^21^, ^22^ Mitochondrial morphology and organization regulate the transport and distribution of mitochondrial content and are critical to maintaining normal mitochondrial function. Through its key role in mitochondrial organization, changes in OPA1 expression affect the stability of the respiratory supercomplexes,^23^ quaternary structures adopted by components of the electron transport chain to carry out cellular respiration.^24^ Cellular studies therefore provide extensive evidence that OPA1 functions to regulate mitochondrial morphology and function. Despite this, it remains unclear as to why RGCs are particularly susceptible to OPA1 disruptions in vivo.

Whilst in vitro models are invaluable for investigating the molecular underpinnings of disease, there are shortcomings in their applicability to studying AOA. The specific architecture of RGCs, with long and heterogeneously myelinated axons, cannot yet be accurately replicated in cell culture.^25^ Moreover, as the progressive loss of visual acuity is a key unifying symptom of AOA, the ability to assess visual function is paramount to both the study of AOA disease pathology and assessing the value of any potential treatment. Studying *OPA1* in vivo has proved challenging as the homozygous knockout (KO) of *OPA1* is developmentally lethal in the existing animal models. *Opa1* ^*−/−*^ mouse embryos appear unable to develop beyond 13.5 days post coitum.^26^ *Drosophila* homozygous KO animals also seem to exhibit embryonic lethality.^27^ It is therefore likely that *OPA1* has an important developmental role, although the precise nature of this is unclear. In order to overcome these limitations, we set out to develop novel in vivo model systems in which to investigate how the loss of *OPA1* contributes to axonopathy and impaired visual function. Here we describe the generation of novel AOA models in both zebrafish (*Danio rerio*) and fruit fly (*Drosophila melanogaster*) using targeted CRISPR‐Cas9 gene editing. We find that the loss of *Opa1* in vivo results in highly conserved disruption of mitochondrial morphology and function. Moreover, the targeted knockout of *Opa1* in zebrafish larvae causes marked visual impairment and mitochondrial damage within the axons of RGCs, recapitulating key characteristics of AOA.

## 2 MATERIALS AND METHODS

### 2.1 Zebrafish breeding and maintenance

Juvenile and adult zebrafish (*Danio rerio*) were raised on a recirculating water system at 28°C with a daily 14‐h lights on, 10‐h lights off cycle. The Tg*(isl2b:mitoeGFP‐2ATagRFPCAAX)* line was originally generated by Prof. F Poulain and Dr. T. Verreet^28^ and kindly provided by the lab of Lieve Moons at Katholieke Universiteit Leuven, Belgium. Larvae were raised in an incubator with a 14‐h light: 10‐h dark cycle at 27°C in E3 media up to 131 h post fertilization (hpf). All larval experiments were performed on animals under 131 hpf (<131 hpf). Animals over 131 hpf were raised under Health Products Regulatory Authority Project AE18982/P186.

### 2.2 Generation of *opa1* crispants and *opa1* ^*−/−*^ zebrafish

The microinjection method has been described previously in Ward et al.^29^ CRISPR/Cas9 genome editing was carried out using Integrated DNA technologies Alt‐R reagents. 36 ng/μL of both CRISPR RNA (crRNA) guides (5′‐CCTGCCAAGGGTTAGTCTTATTC‐3′, 5′‐TGCGGGAAAGACTAGTGTGCTGG‐3′) were heated to 95°C in nuclease‐free duplex buffer along with 67 ng/μL trans‐activating CRISPR RNA (tracrRNA) for 10 min. These solutions were then combined and incubated with 0.5 μg/μL *Streptococcus pyogenes* Cas9 protein at 37°C for 15 min. The resulting mixture was injected into 1–2 cell stage wild‐type (WT) zebrafish embryos. Stable mutants were raised to adulthood, and their offspring screened for germline transmission of a mutant *opa1* allele. One of these mutants was selected and outcrossed with WT animals. Genotyping was conducted as described below.

### 2.3 Genotyping

PCR Primers used for genotyping (Table S1) consisted of a pair of flanking primers and a primer within the deletion region. DNA was extracted from either whole zebrafish larvae or a section of adult caudal fin using Proteinase K (Invitrogen). For sequencing, DNA was extracted from PCR products using the Nucleospin Gel and PCR Clean‐up according to the manufacturer's instructions (Takara Bio). DreamTaq polymerase (Thermo Fisher) was used for all genotyping.

### 2.4 Zebrafish optokinetic response

Zebrafish larvae <131 hpf were immobilized in 9% methylcellulose in E3 media (25 cP, Sigma Aldrich) and placed in the center of a drum rotating at 19 rpm with either 20 (0.02 cycles per degree (cpd)) or 148 (0.2 cpd) vertical black and white stripes. A reduced black/white contrast (20%) drum was also used. All drums used were 3D printed to order by Materialize (Belgium) and the optokinetic response (OKR) assay was conducted as previously described, quantifying the number of saccades.^30^

### 2.5 Zebrafish coiling assay

~24 hpf zebrafish larvae were individually monitored using an Olympus SZX10 light microscope for 30 s. The number of spontaneous tail coiling movements was manually recorded and larvae genotyped as described above.

### 2.6 Zebrafish touch‐startle response

Zebrafish larvae at ~125 hpf were placed in the center of a 60 mm Petri dish filled with E3 media. 3 equidistant circles were drawn on the base of the plate. The tail of the larvae was then touched with a P200 pipette tip, up to 3 times. A score was attributed to the larvae dependent on the number of circles the larvae were able to traverse, with a score of 3 corresponding to a larvae that reached the edges of the plate.

### 2.7 Zebrafish visual motor response (VMR)

48 zebrafish larvae at ~123 hpf were each placed in a single well of a flat‐bottomed 96 well plate with 600 mL E3 embryo media. The plate was then allowed to acclimatize within the Zebrabox system (Viewpoint Life Sciences, Canada) for 30 min prior to the start of the experiment. The activity of each larva was recorded over 100 min, with alternating 20‐min periods of light and darkness as previously described.^31^, ^32^ Settings were: detection sensitivity 20, freeze 5, and burst 25. Activity was calculated using the sum of medium (middur) and high (burdur) activity levels per second. Analysis was conducted using the sum of the average middur and burdur for each animal over the time periods shown in Table 1, in addition to the overall period (6000 s) (Figure 3C).

**TABLE 1:** Time periods analyzed during VMR assays.

Time period	Duration 1 (s)	Duration 2 (s)
Average ON	2400–3599	4800–6000
Average OFF	1200–2399	3600–4799
Pre‐ON	2300–2400	4700–4800
Pre‐OFF	1100–1200	3500–3600
ON Peak	2400–2405	4800–4805
OFF Peak	1200–1205	3600–3605

### 2.8 *Drosophila* maintenance

Fruit flies were maintained at 25°C on a 12‐h light–dark cycle. Generally, animals were maintained on a standard fly food mixture (cornmeal, yeast, dextrose and agar, with tegosept and propionic acid as anti‐fungal agents). To generate neuron‐specific KO of Opa1, the following fly lines were obtained from the Bloomington *Drosophila* Stock Centre (BDSC, https://bdsc.indiana.edu/): Opa1 P{TKO.GS00657} (BDSC 76932) and elavG4;Cas9 (BDSC 67073). Other fly stocks used were UAS‐mito::GFP (BDSC no. 42737)^33^ and *w* ^*1118*^ control (line number 60100, obtained from the Vienna *Drosophila* RNAi Centre, www.vdrc.at).

### 2.9 *Drosophila* survival assay

Virgin female flies of the elavG4, Cas9 genotype were housed with male flies of the Opa1 TKO (II) or a w^1118^ control line (60100). Once wandering instar larvae were observed, the parents were removed. Offspring that had undergone pupation were transferred to a fresh vial, and mortality was recorded every day.

### 2.10 Quantitative PCR

Initial homogenization steps varied based on species. For zebrafish tissue, 10–20 zebrafish larvae were homogenized using a 1 mL syringe in 250 μL of TRIzol reagent (Life Technologies, USA). For *Drosophila* samples, 30 adult heads per sample were then homogenized in 250 μL TRIzol reagent (Life Technologies, USA) with a mortar and pestle in the presence of liquid nitrogen. Total RNA was then purified according to the manufacturer's instructions. 2 μg of this RNA was DNase treated (Sigma‐Aldrich), then cDNA was synthesized using the Superscript III First Strand Kit according to the instructions provided (Invitrogen). Fast SYBR® Green Real‐Time PCR Master Mix (Applied Biosystems™) was used for qPCR on a 7500 FAST real‐time PCR machine (Life Technologies). Rp49 and β‐actin were used as reference genes for *Drosophila* and zebrafish samples, respectively. Primer sequences are included in (Table S1). Relative gene expression was calculated using the −ΔCt method, normalizing mRNA expression from KO animals to control animals.

### 2.11 Proteomic analysis for mass spectrometry

Either 30 adult fly heads or 25 zebrafish larval heads were added to 100 μL of LYSE buffer (PreOmics, Germany) and 45 mg of protein extraction beads (Diagenode, Belgium) and homogenized in an ultrasonic bath for 35 min. The protein concentration of the solution was then determined using a bicinchoninic acid assay (BCA) protein assay kit (Thermo Scientific, USA) according to the manufacturer's instructions. 100 μg of sample protein was made up to a final volume of 85 μL with LYSE buffer. These samples were washed and purified as per the protocol for the iST protein preparation kit (PreOmics, Germany). All samples were then centrifuged at 10 000 rpm for 5 min at 4°C, and 20 μL of sample was taken from the upper fraction for analysis.

Zebrafish samples used a Thermo Scientific (USA) Q Exactive mass spectrometer with a Dionex Ultimate 3000 (RSLCnano) chromatography system for peptide detection. Drosophila samples were analyzed using a TimsTOF Pro mass spectrometer (Bruker, USA) with an Evosep One chromatography system. In both cases, peptides were separated using C18 reversed‐phase columns.

MaxQuant^34^, ^35^ was used to analyze the raw data (version 2.0.3.0 for zebrafish, version 2.4.2.0 for fruit fly samples), incorporating the Andromeda search engine.^36^ MS/MS spectra were matched against the database DANIO_RERIO (46693 entries, 2023_02 release) or DROSOPHILA_MELANOGASTER (22061 entries, 2023_03 release). All searches were performed using default MaxQuant settings. Trypsin was the specified enzyme allowing 2 missed cleavages and a false discovery rate (FDR) of 1% on the peptide and protein levels. Carbamidomethyl (C) was used as a fixed modification, and acetylation (N terminus) and oxidation (M) were used as variable modifications for search purposes.

Perseus software (version 1.6.13.0) was used to analyze label‐free quantitative (LFQ) intensities. Proteins were included in the analysis if 100% of samples in one group (control or loss of *Opa1*) were present. Imputation with values from a normal distribution was used to replace missing values. All remaining values were analyzed by student's *t*‐test analysis with permutation‐based FDR (5%) (*S*_0_ = 1). The dataset was then normalized by *Z*‐score. Additional analysis of differentially expressed (DE) proteins was conducted using Ingenuity Pathway Analysis (IPA) (Qiagen, Germany). IPA analysis was performed using orthologous human proteins.

### 2.12 Histology and immunomicroscopy in *Drosophila*

For larval neuromuscular dissections, third‐instar larvae were dissected in chilled Ca^2+^‐free HL3 solution and fixed in 4% formaldehyde solution for 30 min as previously described.^37^ Dissected larvae were mounted onto slides with vectashield (VectorLabs, USA) and imaged with an Olympus FluoView FV100 confocal microscope. Images were acquired using a 60×/1.5 NA objective and using the FV10‐ASX version.04.01 software.

### 2.13 Mitochondrial morphology analysis in *Drosophila*

Images were analyzed by first adjusting the threshold in ImageJ to enable the largest number of individual mitochondria to be visible. The image was then processed to make binary. The particle analysis tool was then used to measure “bounding rectangles” to measure mitochondria length and “shape descriptors” to calculate mitochondrial circularity. Images were manually assessed to remove merged or incorrectly partitioned mitochondria.

### 2.14 Histology and immunomicroscopy in zebrafish

For light microscopy, zebrafish larvae ~124 hpf were euthanized on ice and fixed as described briefly.^38^ Briefly, larvae were initially fixed in a solution of 2% paraformaldehyde and 2.5% glutaraldehyde in 0.1 M Sorenson phosphate buffer pH 7.3 for at least 2 days. Subsequently, samples were fixed in a solution of 1% osmium tetroxide, then dehydrated using a series of increasing ethanol gradients and finally propylene oxide. Samples were finally incubated in Epon 812 resin overnight, before fixing in fresh resin overnight at 60°C. The resin blocks were then cut into 1000 nm thick sections using a Leica ultramicrotome EM UC6, stained using toluidine blue, and adhered onto slides using DPX mountant.

For immunomicroscopy, zebrafish larvae ~124 hpf were fixed in a solution of 4% paraformaldehyde at 4°C overnight. Samples were washed three times for 5 min with phosphate buffered saline (PBS). Larvae were bleached (0.18 M KOH and 3% H_2_O_2_ in ddH_2_O) for 20 min to remove pigment. Larvae were then washed three times with PBS‐T (PBS, 1% Triton‐X). The larvae were permeabilised with 150 mM Tris–HCl (pH 8.8) at room temperature for 5 min, before being incubated at 70°C for 15 min in a water bath. After removal from the water bath, the samples were washed twice for 10 min in PTw (PBS, 0.1% Tween), then twice for 5 min in ddH_2_O. The samples were further permeabilised by incubation in acetone at −20°C for 20 min. The larvae were again washed in the same manner. Following this, the samples were incubated in blocking buffer (10% goat serum [Sigma, USA], 0.8% Triton‐X, 1% BSA (Sigma, USA) and 1% DMSO in PTw) for 3 h. The samples were then incubated for 2 days in Hoechst (1:100, Thermo Scientific, USA) in incubation buffer. Subsequently, the samples were washed in PBS‐TS for 1 h 3 times, then twice for 1 h with PTw. The larvae were then mounted in Aqua/ Polymount (Polysciences, USA) and were imaged using a ZEISS Laser Confocal Scanning Microscope 800 (with Airyscan deconvolution).

### 2.15 Electron microscopy

Zebrafish larvae at ~124 hpf were euthanized and fixed as described for LM above. Ultrathin sections of 80 nm were collected using a Leica ultramicrotome EM UC6 onto 200 mesh copper grids and stained with 2% uranyl acetate for 20 min and 3% lead acetate for 5 min. Stained samples were imaged using a Tecnai G2 12 BioTWIN at 120 KeV.

### 2.16 Mitochondrial morphology analysis in zebrafish

Widths of retinal layers were measured by taking 20 measurements across the layer of the larval eyes using Image J. An average was calculated, and this value was used for statistical analysis. In wholemount larvae, the transgenic line Tg*(isl2b:mitoeGFP‐2ATagRFPCAAX)* labeled the mitochondria and cell membranes of RGCs with eGFP and RFP respectively. In these samples, the boundaries of the inner plexiform layer (IPL) and ganglion cell layer (GCL) were defined by the absence (for the IPL) or presence (for the GCL) of nuclei in the cell layer when labeled with Hoechst stain. For quantifying fluorescence, the mean gray value function in ImageJ was used to quantify fluorescence intensity of the IPL immediately adjacent to the optic nerve. Ultrastructural analysis of mitochondrial morphology was performed on TEM images taken of RGC axons within the optic nerve. The oval brush tool in ImageJ was used to manually trace around and define bounding mitochondrial area. From this the Shape Descriptors option in the ImageJ/Analyze menu was used to define: (i) length of longest mitochondrial axis, (ii) mitochondrial circularity and (iii) mitochondrial area (Figure S1A). Mitochondria defined as having “Internal structure evident” or “devoid of internal structure” (as demonstrated in Figure S1B) were manually counted. To measure the volume of cristae as a proportion of the mitochondrial matrix, the freehand selection tool within ImageJ was used to manually draw around the perimeter of the matrix, and the area of this shape measured. Each crista was then outlined and their area measured in the same manner. The total area of all cristae was calculated as a percentage of total matrix area (Figure S1C). For visualization of distribution of mitochondrial morphological metrics across all samples, perseus 1.6.15.0 was used. Data was *z*‐score normalized followed by hierarchical clustering and principal component analysis. Hierarchical clustering used euclidean distance means, average linkage and k‐means clustering for both the rows and column tree.

### 2.17 Seahorse analysis

Buffer‐injected and crispant *Opa1* larvae were generated as described earlier. At either 0 or 1 day post fertilization (dpf), the embryos were placed into 50 mL tubes filled with E3 media and placed inside an insulated box with a heat pack. The larvae were then shipped overnight to the University of Montpellier. At <131 hpf, Seahorse assays were conducted as described previously.^39^

### 2.18 Statistical analysis

Unless stated otherwise, statistical analysis was carried out using GraphPad Prism software (Dotmatics, USA). Experiments with 2 independent groups were either analyzed using an unpaired 2‐tailed *t*‐test or Mann–Whitney test in the event that the data did not follow a Gaussian distribution. For experiments with more than 2 independent groups, a one‐way ANOVA with Tukey multiple comparison test was performed for data following a Gaussian distribution, or a Kruskal‐Wallis test followed by a Dunn's multiple comparison test in the case that the dataset did not follow a Gaussian distribution. All experimental data is presented as mean ± standard deviation (SD). Statistical significance was ascribed to *p* values of *p* < .001 (****), *p* < .001 (***), *p* < .01 (**), and *p* < .05 (*).

## 3 RESULTS

### 3.1 Generation of fish and fly Opa1 loss of function models

**FIGURE 1:**
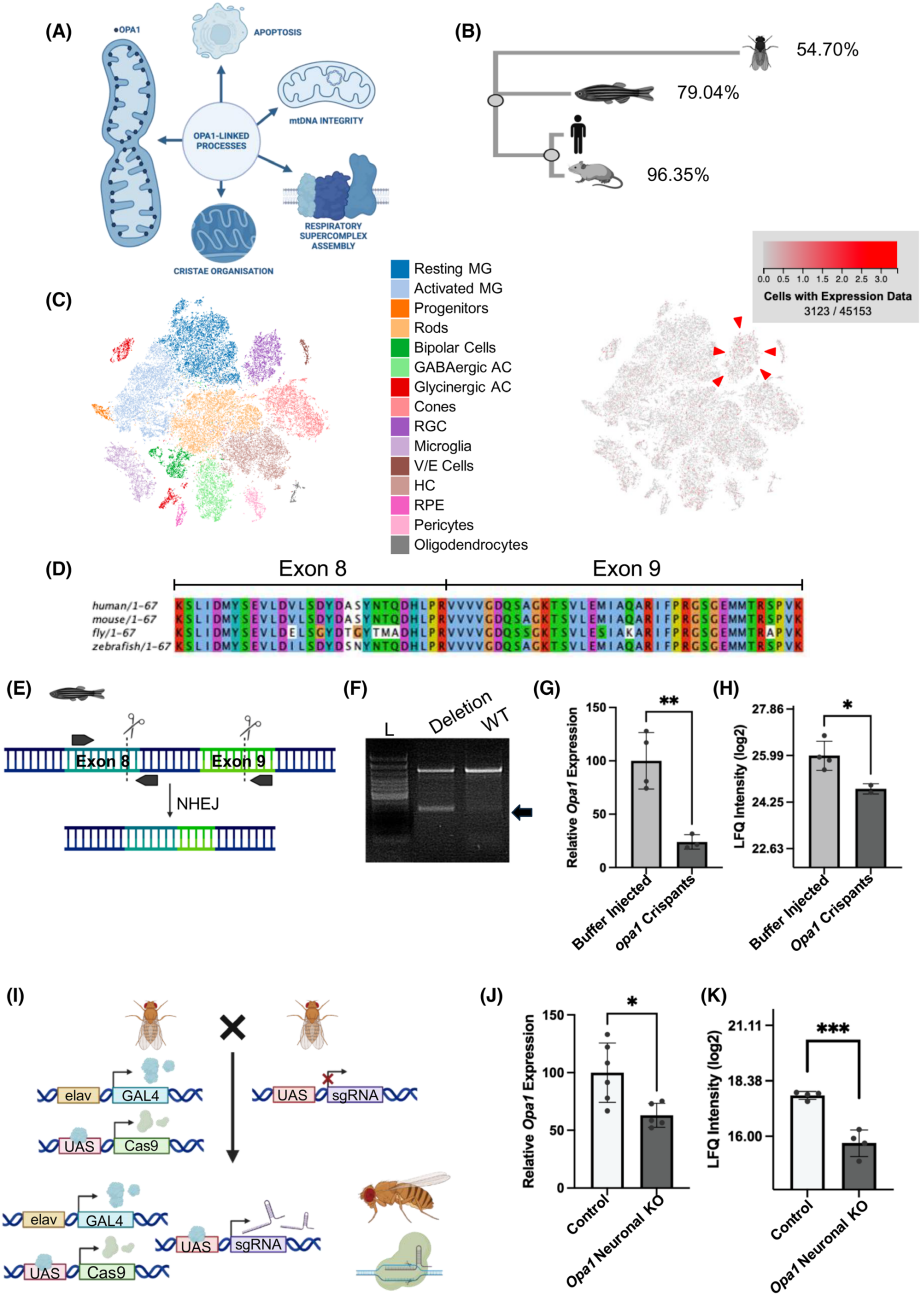
Generation and validation of 2 in vivo models of Opa1 deficiency. (A) Schematic illustrating the localisation and range of functions of Opa1. (B) Dendrogram showing similarity between human, mouse, zebrafish and *Drosophila* Opa1 proteins generated following Clustal alignment. Percentage identities of the amino acid sequence of OPA1 of the respective model organisms to the canonical isoform of human OPA1 are shown. (C) Expression of *opa1* in the zebrafish retina from online scRNA‐seq data https://proteinpaint.stjude.org/F/2019.retina.scRNA.html. Red marks cells with *opa1* expression (RGC indicated with red arrowhead). (D) Amino acid sequence of exons 8 and 9 of the *Opa1* gene in human, mouse, zebrafish and *Drosophila*. (E) Schematics illustrating the guide locations of the guide pair (scissors) and PCR primers (arrows) used to generate *opa1* editing in zebrafish. (F) A representative PCR genotyping electrophoresis gel from individual larvae injected with opa1 guides with (Deletion) or without (WT) a deletion event. NHEJ produces a deletion band of approximately 350 bp (black arrow). (G, H) Graphs represent mean ± standard deviation (SD) mRNA (G) and protein (H) Opa1 expression in buffer injected controls and *opa1* crispant larvae. Statistical analysis consists of two‐tailed *t*‐tests. *n* = 3–4. (I) Schematic illustrates the crossing strategy to generate neuronal *Opa1* KO *Drosophila*. (J, K) Graphs represent mean ± standard deviation (SD) mRNA (J) and protein (K) Opa1 expression in progeny of *elav‐GAL4.UAS‐Cas9* crossed to either *w* ^*1118*^ (control) or *Opa1 sgRNA* (*Opa1* neuronal KO) flies. Statistical analysis consists of two‐tailed *t*‐tests. *n* = 4–6.

Opa1 is expressed in the inner mitochondrial membranes of all metazoans (Figure 1A). However, the investigation of Opa1 function in living animals has been hampered by early developmental lethality caused by homozygous disruption of the *Opa1* gene.^26^, ^27^, ^40^ Therefore, we decided to use CRISPR‐based technologies to assess neuronal‐specific disruption of Opa1 in flies and deletion of the *Opa1* gene in zebrafish—species which show high protein conservation with the human OPA1 protein (Figure 1B).

As *OPA1* mutations are linked to impaired vision and retinal ganglion cell (RGC) defects in humans,^2^, ^41^ we assessed the transcript expression pattern of *opa1* in the zebrafish retina using an existing scRNAseq dataset^42^ (Figure 1C). Notably, zebrafish *opa1* does not appear to be enriched in RGCs, displaying low mRNA expression in all retinal cell clusters, including the RGC population (Figure 1C). The crispant approach provides an opportunity to generate targeted gene mutations without the generation time needed for germline transmission.^43^ We strategically targeted *opa1* exons 8 and 9, a region coding amino acid (aa) sequences with high conservation (>86%) across species (Figure 1D) and encompassing the beginning of the GTPase domain that is integral for many aspects of Opa1 function and a major site for pathogenic mutations in patients.^10^ crRNAs targeting Cas9 to exons 8 and 9 were designed to induce a ~930 bp deletion in the *opa1* gene by non‐homologous end joining (NHEJ) (Figure 1E). The zebrafish *opa1* crispant genotype was confirmed by PCR wherein a large genomic deletion was present in 90.3% of genotyped F0 samples (*n* ≥ 600 larvae) (Figure 1F). Validation of the *opa1* crispant model included evidence of significantly reduced opa1 mRNA (Figure 1G) and Opa1 protein (Figure 1H) expression, confirming expression of endogenous zebrafish Opa1 has been disrupted.

Organization of the *Drosophila* visual system is very different from that of humans or zebrafish. Nevertheless, flies offer great potential to investigate mechanisms of axonal degeneration more generally. Given that ubiquitous loss of Opa1 results in embryonic lethality in *Drosophila*,^27^ the UAS‐GAL4 system was used to target disruption of Opa1 only in neurons. Cas9 and sgRNAs targeting Opa1 were expressed using the neuron‐specific elav‐GAL4 line, resulting in F1 flies in which Opa1 is mutated in neurons only (Figure 1I). Expression of Opa1 mRNA (Figure 1J) and Opa1 protein (Figure 1K) are significantly reduced in *Drosophila* heads, which are enriched for neurons, confirming that this approach results in *Drosophila* in which endogenous Opa1 has been disrupted.

### 3.2 Zebrafish Opa1 loss of function models display severely impaired visual behavior

**FIGURE 2:**
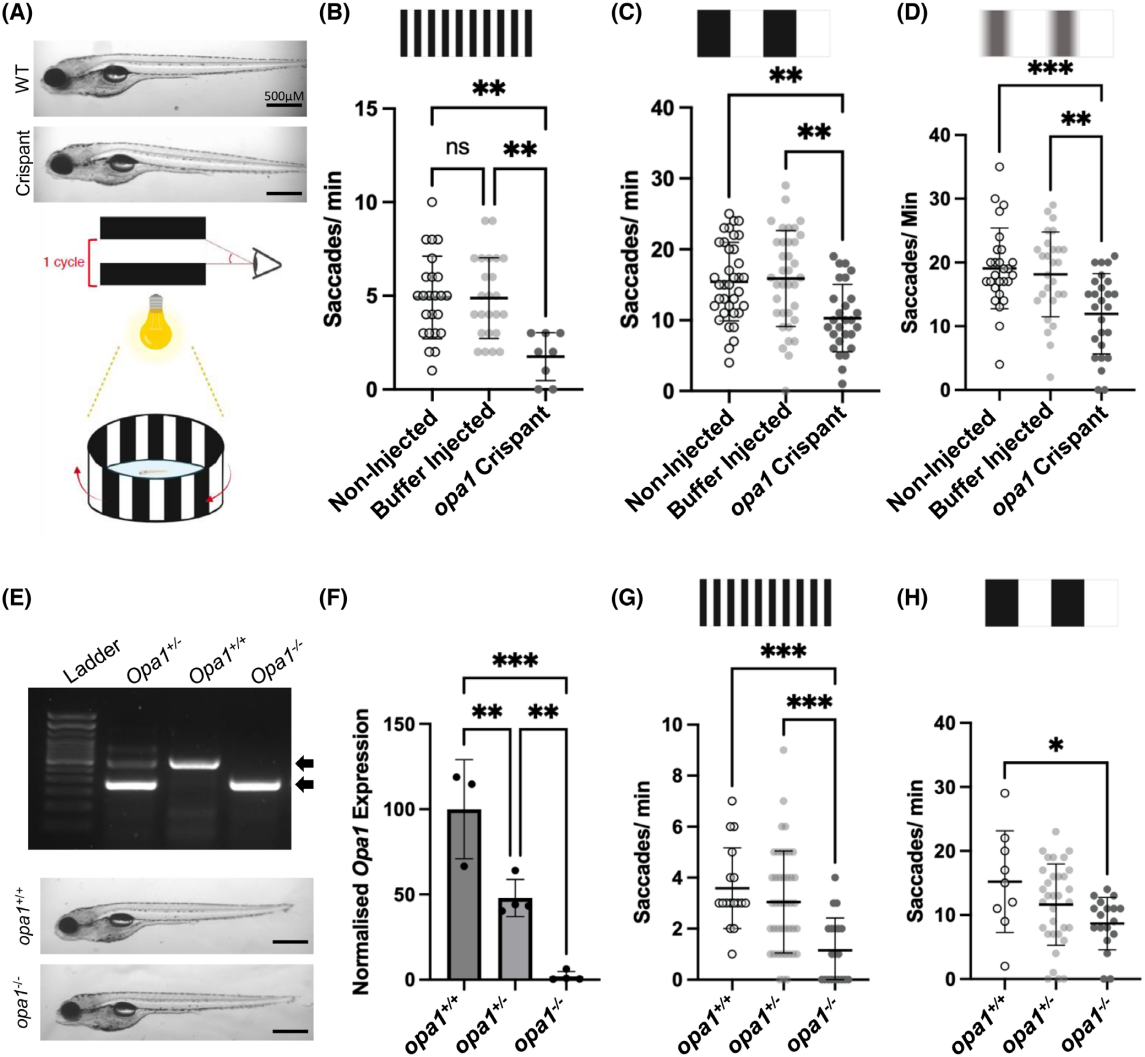
Loss of *Opa1* impairs visual function in zebrafish. (A) Schematic of OKR assays which were used to assess visual function in <131 hpf larvae. No gross morphology differences are distinguished between *opa1* crispants and buffer injected controls. (B–D) Visual acuity responses of <131 hpf *opa1* crispants and controls to drums with 0.2 (B) and 0.02 (C) cycles per degree (cpd) and 0.2 cpd with reduced (20%) black/ white contrast (D). (E) A representative PCR genotyping electrophoresis gel from *opa1* ^+/+^, *opa1* ^+/−^ and *opa1* ^−/−^ larvae. *opa1* mutation produces a 932 bp deletion band. No gross morphological differences were observed between *opa1* ^*−/−*^ animals and *opa1* ^*+/+*^ siblings. (F) qPCR shows a significant reduction of opa1 transcript (normalized to B‐Actin) in both *opa1* ^−/−^ and *opa1* ^*+/−*^ animals. (G, H) Visual acuity responses of <131 hpf *opa1* ^+/+^, *opa1* ^+/−^ and *opa1* ^−/−^ larvae to drums with 0.2 (G) and 0.02 (H) cycles per degree. All graphs represent mean ± standard deviation (SD) saccades per minute. Statistical analysis consists of one‐way ANOVA and Tukey's multiple comparisons tests.

To assess the impact of Opa1 disruption on visual function, we employed the optokinetic response (OKR) assay.^30^, ^44^ Larvae will preferentially move their eyes in the direction of perceived motion—a movement known as a saccade (Figure 2A). Crispants with *opa1* deletions confirmed via PCR genotyping demonstrated significant reductions in the number of saccades with a drum of 0.2 (Figure 2B) or 0.02 cycles per degree (cpd) (Figure 2C) compared to both their non‐injected or buffer‐injected siblings. The reduction in saccades was greater for the thinner 0.02 cpd stripe pattern, with a 33.29% decrease compared to 64.43% for the 0.2 cpd drum. *opa1* crispants also demonstrated a significant reduction (37.28%) in contrast sensitivity when the OKR was conducted using a drum with reduced (20%) black contrast (Figure 2D).

Some *opa1* crispants were raised to adulthood, and an individual carrying a germline *opa1* mutation was identified by genotyping its offspring. DNA sequencing confirmed these *opa1* CRISPR mutants possessed a deletion of 932 bp spanning exons 8 and 9 of the endogenous *opa1* gene (Figure 2E). The resulting frameshift mutation is predicted to generate a premature STOP codon, resulting in a truncated Opa1 protein of 304 aa compared to the 1034 aa of the longest Opa1 isoform (Figures S2 and S3). Any resulting truncated Opa1 protein produced (either l‐Opa1 or s‐Opa1) would lack the GTPase, dynamin, and GTPase effector domains (Figure S2B) required for regulating mitochondrial morphology.^10^ o*pa1* ^*−/−*^ homozygotes were viable to <131 hpf but were absent upon colony genotyping at 2 months old. <131 hpf larvae from the germline knockout revealed a 52% and 98% decrease in *opa1* transcript in *opa1* ^*+/−*^ and *opa1* ^−/−^ larvae compared to wildtype, respectively (Figure 2F). *opa1* ^*−/−*^ larvae were also subjected to OKR assays and demonstrated a significant reduction (67.95%, 43.06%%) in visual acuity with both the 0.2 cpd (Figure 2G) and 0.02 cpd drums (Figure 2H). Together, these findings reveal that *opa1* disruption in zebrafish causes significant visual impairment, confirming the generation of a novel model of OPA1‐associated AOA.

### 3.3 Characterization of non‐visual behavioral phenotypes in Opa1 loss of function flies and fish

**FIGURE 3:**
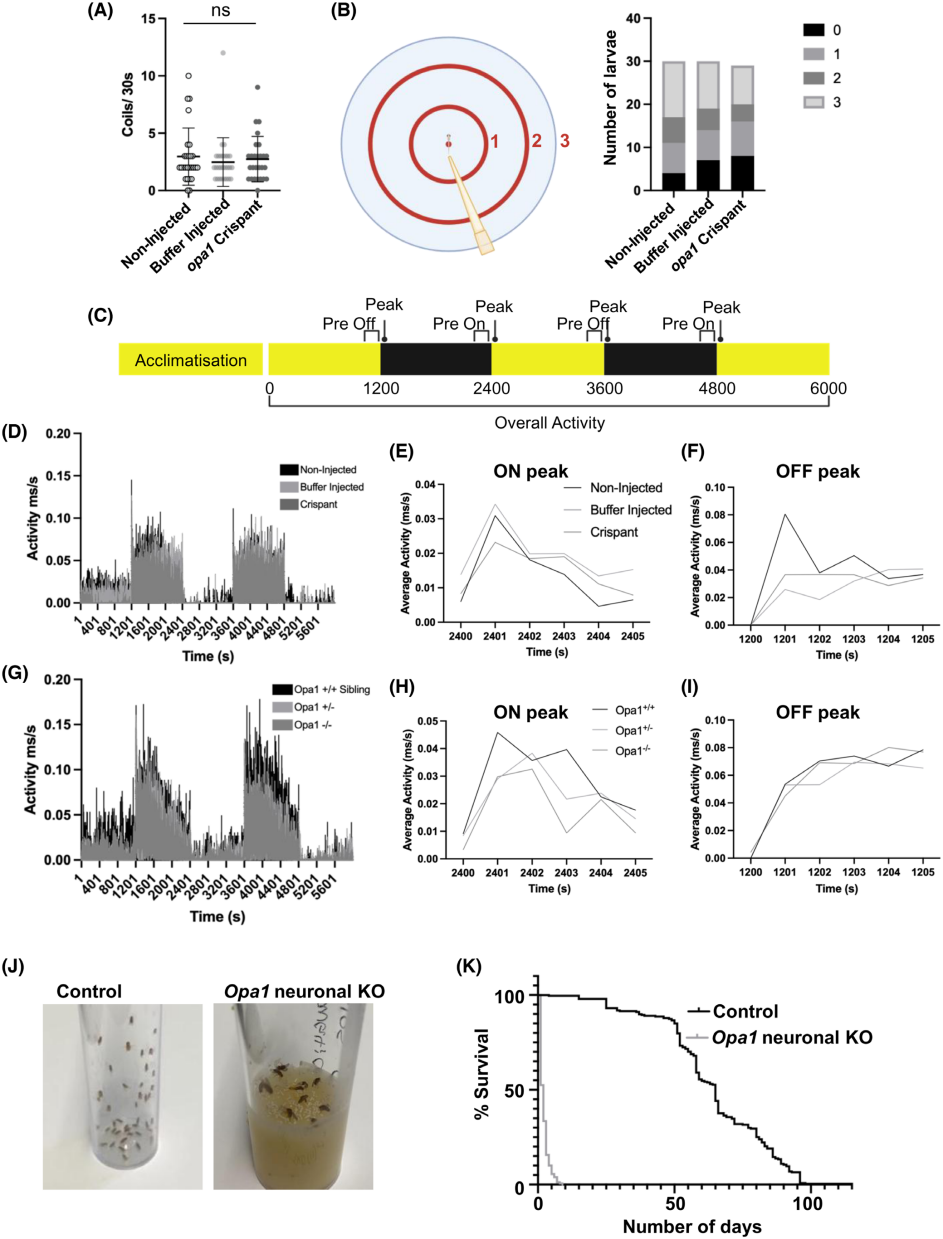
Nonvisual phenotypes in Opa1 deficiency models. (A) Graph represents mean ± standard deviation (SD) number of spontaneous tail coiling movements in 30 s in 24 hpf larvae. No significance (ns) detected by Krustal‐Wallis test. (B) Schematic illustrating the apparatus used for a touch response assay. Graph represents the number of 125 hpf larvae that swim the indicated distance from the starting point in response to a gentle tap on the tail. (C) Schematic illustrating the periods used for activity recording in VMR assays. Black and yellow bars indicate dark and light conditions, respectively. (D–I) Results of VMR assays involving opa1 loss of function zebrafish. No significance was detected by Krustal Wallis test. Activity traces showing activity over the course of an entire VMR assay (100 min) in <131 hpf *opa1* crispant (D) and *opa1* ^−/−^ (G) larva compared to relevant controls. Average ON peak activity for both on peaks in *opa1* crispant (E) and *opa1* ^−/−^ (H) larvae and controls. Average OFF peak activity for both peak periods in opa1 crispant (F) and *opa1* ^−/−^ (I) larvae and controls. (J) Stills from videos of progeny of *elav‐GAL4.UAS‐Cas9* crossed to either *w* ^*1118*^ (control) or *Opa1 sgRNA* (*Opa1* neuronal KO) flies. (K) Graph represents % survival of *Opa1* neuronal KO and control flies. *n* = 179–247 flies per genotype. *p*‐value < .0001 determined by Log‐rank (Mantel‐Cox) test.

As a subset of individuals with AOA have extra‐ocular neuronal and motor deficits, additional behavioral assays were conducted to determine if comparable dysfunctions were present within the novel Opa1 loss of function models generated. In zebrafish *opa1* crispant larvae, coiling (Figure 3A) and touch startle (Figure 3B) responses were unchanged from buffer injected controls, indicating no gross neurodevelopmental defects. Locomotor behavior was assessed by the visual motor response (VMR) assay, revealing that *opa1* crispant animals did not demonstrate any significant difference in overall activity compared to buffer or non‐injected siblings (Figure 3C). VMR activity is typically highest immediately after changes in light conditions, from “ON” to “OFF” or vice versa. The 5‐s timeframe following the light change is analyzed as the “ON” or “OFF” peak, when activity should be highest.^32^ Again, the maximum VMR ON and OFF activity was not significantly changed in *opa1* crispant larvae (Figures 3D–F and S4). Likewise, *opa1* ^*−/−*^ CRISPR germline knockout larvae did not demonstrate significantly altered overall or maximal activity compared to heterozygous or WT siblings (Figures 3G–I and S5). Together, these findings suggest that loss of Opa1 does not significantly disrupt locomotion in zebrafish larvae.

In contrast to our findings in Opa1 disrupted zebrafish larvae, neuron‐specific *Opa1* knockout *Drosophila*, while viable, display obvious developmental and motor defects. Neuronal *Opa1* knockout flies frequently failed to inflate their wings and rarely demonstrated the ability to walk at all, often displaying uncontrolled seizure‐like movements of their limbs (Figure 3J and Video S1). These neuronal *Opa1* knockout flies had a significantly reduced lifespan, with a median survival of just 2 days post‐eclosion, compared to 65 days in control flies (Figure 3K). This demonstrates an essential role for Opa1 in *Drosophila* neurons post‐development. Interestingly, while *opa1* ^−/−^ zebrafish larvae are viable to 131 hpf, they perish before 2 months old, indicating that Opa1 expression is similarly required for survival in adult zebrafish.

In summary, disruption of Opa1 in zebrafish specifically replicates the loss of visual acuity seen in patients, without displaying broad motor symptoms. *Drosophila* neuron‐specific Opa1 knockout has more severe impairments, exhibiting reduced mobility and very early mortality.

### 3.4 Proteins associated with mitochondrial dysfunction are a conserved feature of loss of Opa1 models

To understand the effect of disrupted Opa1 expression in vivo, we used unbiased proteomic analysis in our knockout models of AOA.

**FIGURE 4:**
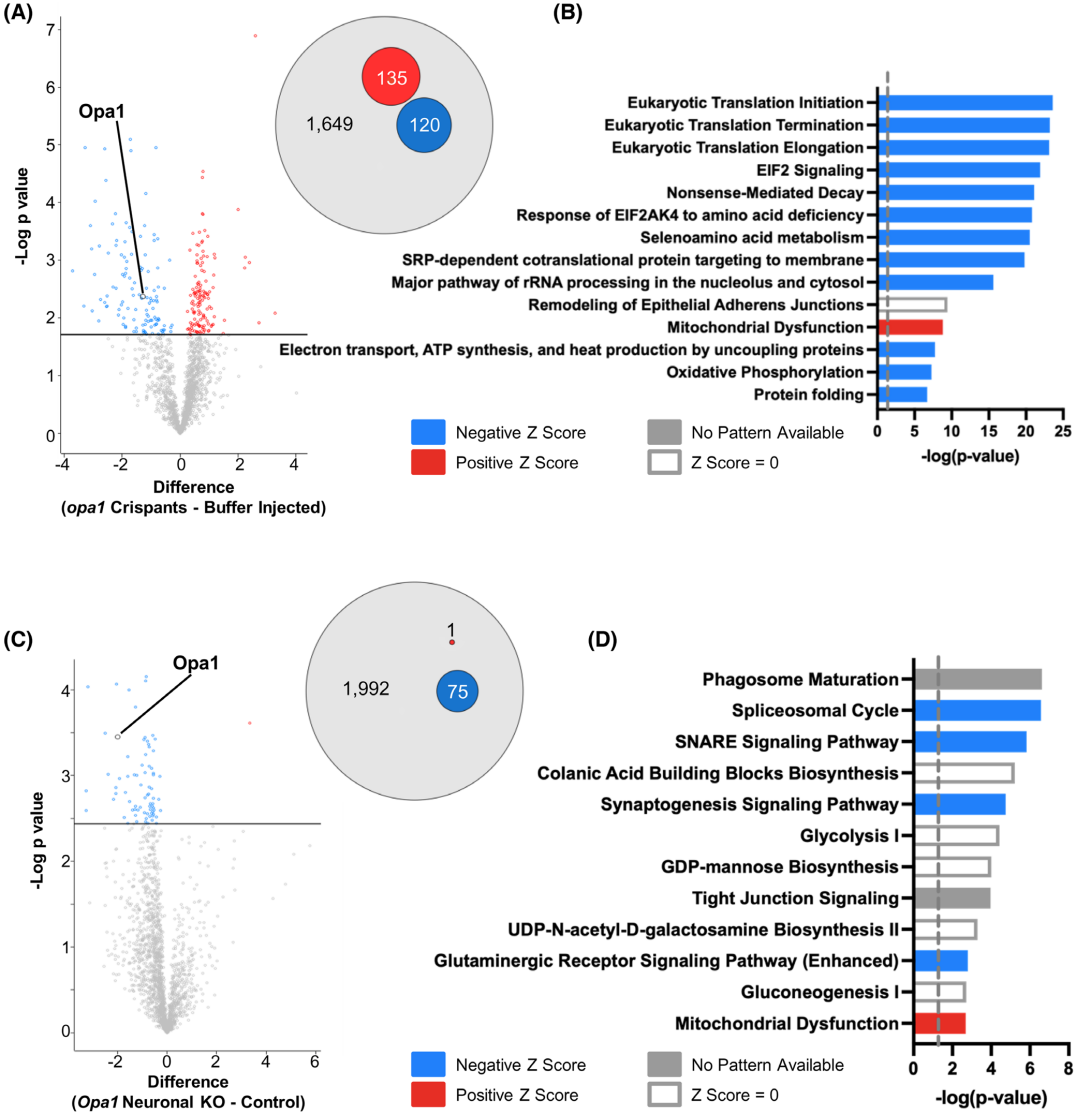
Loss of *Opa1* causes disruption of mitochondrial proteins in both *Drosophila* and zebrafish. (A) Volcano plot depicts the differentially expressed proteins between opa1 crispants and buffer injected controls. The proteins highlighted in red represent the proteins with a Student's *t*‐test difference ≥0.5. The proteins highlighted in blue represent those with a Student's *t*‐test difference ≤−0.5. Opa1 is highlighted. Venn diagram (right panel) shows proportion of up‐ (red) and down‐ (blue) regulated proteins between *opa1* crispants and buffer injected controls. (B) Ingenuity pathway analysis (IPA) of differentially expressed proteins identified by proteomic analysis of *opa1* crispants and buffer injected controls, showing the most enriched pathways identified. (C) Volcano plot depicts the differentially expressed proteins between progeny of elav‐GAL4.UAS‐Cas9 crossed to either *w* ^*1118*^ (control) or *Opa1 sgRNA (Opa1* neuronal KO) flies. The proteins highlighted in red represent the proteins with a Student's *t*‐test difference ≥0.5. The proteins highlighted in blue represent those with a Student's *t*‐test difference ≤−0.5. Opa1 is highlighted. Venn diagram (right panel) shows proportion of up‐ (red) and down‐ (blue) regulated proteins between *Opa1* neuronal KO and controls. (D) Ingenuity pathway analysis (IPA) of differentially expressed proteins identified by proteomic analysis of *Opa1* neuronal KO and controls, showing the most enriched pathways identified.

In zebrafish, heads of *opa1* crispant or buffer‐injected larvae at approximately 123 hpf were analyzed due to the technical constraints of isolating RGCs or the optic nerve. 3207 proteins were initially identified with 1649 proteins remaining following filtering for proteins only identified in all samples from either genotype. Principal component analysis (PCA) on the proteomic datasets showed distinct clustering between *opa1* crispant samples and buffer‐injected controls (Figure S6). After Student's *t*‐test analysis with permutation‐based false discovery rate (FDR), 255 proteins were significantly differentially expressed (DE) with 135 increased and 120 decreased proteins in *opa1* crispants relative to buffer‐injected animals (Figure 4A) (Data are available via ProteomeXchange with identifier PXD059584).

Ingenuity pathway analysis (IPA) using human orthologs of the differentially expressed (DE) proteins was conducted to identify biological pathways significantly altered by the loss of Opa1 (Figure 4B). In zebrafish, many pathways exhibiting the greatest changes were linked to the process of *translation*. eIF2 was highlighted specifically in this context, which is a crucial point of protein synthesis regulation through the integrated stress response.^45^ Other processes linked to protein synthesis including *selenoamino acid metabolism* and *ribosomal RNA processing* were also predicted to be inhibited in *opa1* crispant zebrafish. Together this suggests that protein production is highly dysregulated when Opa1 is disrupted.

In *Drosophila*, 4 samples from either *Opa1* neuronal KO flies or controls were subjected to proteomic analysis, with 30 heads per sample. PCA again indicated separation between the *Opa1* neuronal KO and control samples (Figure S6), 2 of the neuron‐specific KO samples were more similar to the controls than the other mutants. In total, 1992 proteins were identified, of which 75 were downregulated and 1 was upregulated in *Opa1* neuronal KOs compared to controls following statistical analysis (Figure 4C) (Data are available via ProteomeXchange with identifier PXD059645). IPA of DE proteins identified in *Drosophila* revealed large changes in pathways linked to apoptosis and decreased neurotransmission, including phagosome maturation, the soluble *N*‐ethylmaleimide‐sensitive factor attachment protein receptor (SNARE) and synaptogenesis signaling pathways (Figure 4D). These are indicative of broad neuronal dysfunction and likely neuronal cell death, in keeping with the observation that ~50% of the *Opa1* neuronal KO flies died within 24 h.

**FIGURE 5:**
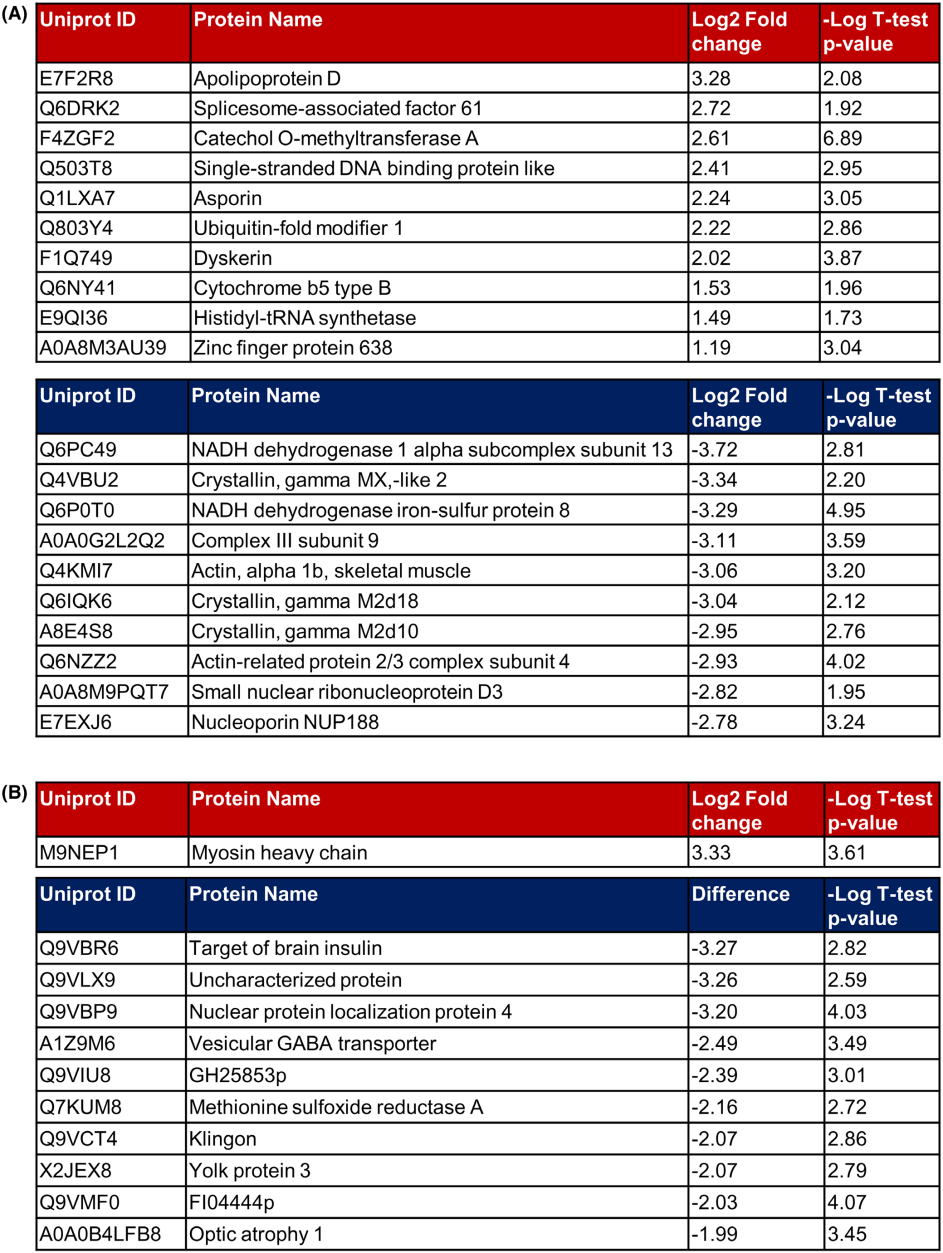
Most significantly altered proteins identified by proteomic profiling in Opa1 loss of function models. (A) Tables show the 10 most up‐ (upper panel) and down‐ (lower panel) regulated proteins in *opa1* crispants compared to buffer‐injected controls. (B) Tables show the 10 most up‐ (upper panel) and down‐ (lower panel) regulated proteins in progeny of elav‐GAL4.UAS‐Cas9 crossed to either *w* ^*1118*^ (control) or *Opa1 sgRNA* (*Opa1* neuronal KO) flies.

Notably, enhanced mitochondrial dysfunction featured prominently within the pathway analysis for both loss of Opa1 models, through a decrease in oxidative phosphorylation and an increase in ‘mitochondrial dysfunction’ pathways (Figure 4B,D). This included components of the electron transport chain, specifically complexes I and III, which were significantly decreased in both loss of Opa1 models. Additionally, we detected a loss of proteins involved in mitochondrial calcium signaling and an increase in mitochondrial heat shock proteins. We also assessed the proteins with the largest fold changes (up to 8‐fold) in expression between the Opa1 models and controls (Figure 5). At an individual protein level, only 5 orthologous proteins are significantly DE in both our loss of function Opa1 zebrafish and fly models. Strikingly, Opa1 is significantly down regulated in both models compared to their respective controls (Figure 1H,K). No change in expression was detected for the orthologs of YMEL1L or OMA1 that regulate cleavage of Opa1 (Table S2). Consistent with the pathways identified by IPA, some of the largest individual expression changes identified were in proteins involved in mitochondrial function including components of the electron transport chain (NADH dehydrogenase 1 alpha subcomplex subunit 13, NADH dehydrogenase iron–sulfur protein 8 and cytochrome b5 type B). Due to the prominence of mitochondrial proteins in the proteomic datasets, and previous observations of mitochondrial abnormalities associated with *Opa1* loss, we decided to more closely examine changes in mitochondrial morphology in the fly and zebrafish knockout models.

### 3.5 Ganglion cell and photoreceptor mitochondria morphology, but not retinal cell lamination, are severely disrupted in loss of function Opa1 models

**FIGURE 6:**
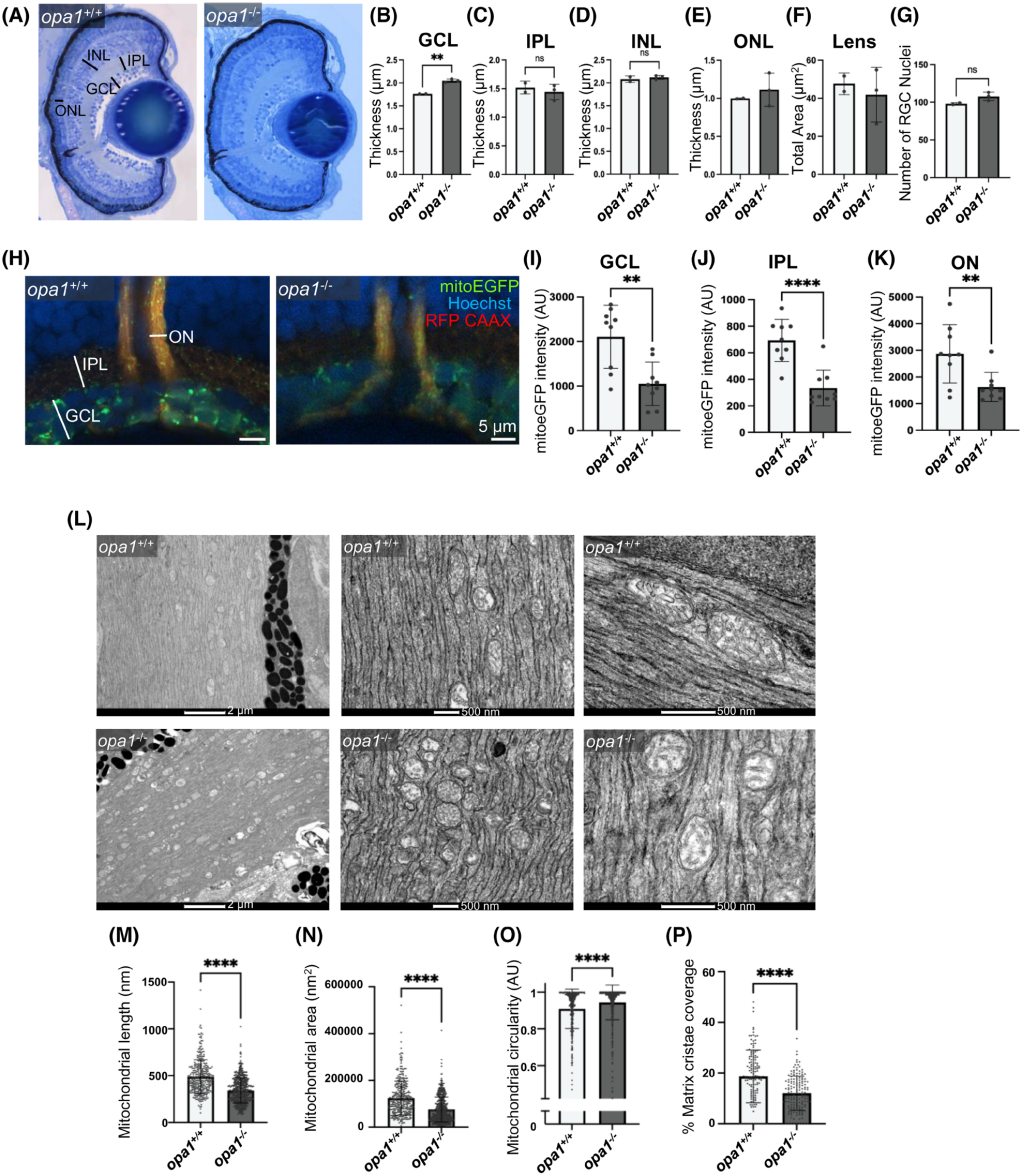
Loss of Opa1 disrupts mitochondrial morphology within RGC axons in zebrafish. (A–F) Cellular lamination is comparable in opa1^−/−^ larvae and opa1^+/+^ siblings. (A) Representative images of toluidine blue stained retinal sections from <131 hpf zebrafish. Retinal layers indicated. Graphs represent mean ± SD thickness in μm of GCL (B), IPL (C), INL (D), ONL (E) and total area (μm^2^) of the Lens (F). (G) Graph represents mean ± SD number of RGC nuclei. (H–K) mitoEGFP staining is significantly decreased in *opa1* ^*−/−*^ larvae compared to *opa1* ^*+/+*^ siblings. (H) Representative confocal image of wholemount retinas of <131 hpf *Tg(isl2b:MitoeGFP‐2ATagRFPCAAX)* zebrafish. Graphs represent mean ± standard deviation (SD) mitoEGFP intensity within the GCL (I), IPL (J) and ON (K). Statistical analysis throughout consists of two‐tailed *t*‐tests. (L–P) Mitochondrial organization is significantly disrupted in *opa1* ^*−/−*^ larvae compared to *opa1* ^*+/+*^ siblings. (L) Representative electron microscopic images of the optic nerve of <131 hpf larval zebrafish. Graphs represent mean ± SD length of longest mitochondrial axis (M), total mitochondrial area (N) mitochondrial circularity (O) and % mitochondrial matrix occupied by cristae (P). Graphs show data from individual mitochondria with statistical analysis performed by Mann Whitney tests (*n* = 226–345).

Our analysis revealed that Opa1 disruption attenuates visual, but not motor, function in <131 hpf zebrafish larvae, so we sought to determine whether loss of Opa1 impairs development of retinal cell layers. Quantification of the thickness and area of retinal layers indicated no consistent differences in thickness between *opa1* mutant zebrafish and their controls (Figures 6A–F and S7A–F). While a thicker ganglion cell layer (GCL) was detected in *opa1* ^*−/−*^ larvae compared to *opa1* ^*+/+*^ siblings (Figure 6B), we believe that this is attributable to fixation artifacts on the samples, specifically tearing of the sections between the lens and GCL, which was present in the *opa1* ^*−/−*^ samples but not in *opa1* ^*+/+*^. In support of this, the number of ganglion cell nuclei was not significantly altered between *opa1* ^*−/−*^ larvae and their *opa1* ^*+/+*^ siblings within the same sections (Figure 6G) and analysis of fluorescently labeled GCLs within independent samples revealed no difference in the thickness of the GCL between *opa1* ^*−/−*^ larvae and their *opa1* ^*+/+*^ siblings (Figure S7A–E). Furthermore, the thickness of the GCL was not significantly altered between *opa1* crispant larvae and their buffer injected siblings (Figure S7G). Taken together, this suggests that loss of Opa1 does not disrupt cellular lamination in <131 hpf zebrafish.

As the proteomic results had identified many mitochondrial proteins that are differentially expressed in the heads of *opa1* crispants, we examined mitochondrial morphology within RGCs when Opa1 is disrupted. The *opa1* ^*−/−*^ line was bred with the transgenic line Tg*(isl2b:mitoeGFP‐2ATagRFPCAAX)* in which eGFP is fused to the mitochondrial targeting signal of the zebrafish cox8a gene, a component of complex IV within the inner mitochondrial membrane.^28^ This line enables fluorescent imaging of mitochondria with RGCs (Figure 6H). Quantification of average fluorescence within the RGC layer (Figure 6I), inner plexiform layer (IPL) (Figure 6J), and optic nerve (ON) (Figure 6K) all demonstrated a significant reduction in eGFP within *opa1* ^*−/−*^ larvae compared to *opa1* ^*+/+*^ siblings. Given the localisation of eGFP within the transgenic construct to the inner mitochondrial membrane, the reduction in eGFP signal may reflect a loss of mitochondria generally, perhaps due to increased mitophagy or decreased mitochondrial biogenesis, or disruption of the inner mitochondrial structure. To explore this further, we performed electron microscopy to examine changes in the morphology and organization of individual mitochondria within RGC axons within the optic nerve (Figures 6L and S8). Mitochondria were not readily identified within autophagic vesicles, nor was there a noticeable loss of mitochondria within *opa1* ^*−/−*^ axons, suggesting that neither mitophagy nor mitochondrial biogenesis is significantly disrupted in RGC axons at this time in response to the loss of *opa1* ^*−/−*^. The mitochondria in *opa1* ^*−/−*^ larval RGC axons are significantly shorter, have a smaller area, and are more rounded (Figures 6M–O and S9) when compared to their *opa1* ^*+/+*^ siblings. Considering the role of Opa1 in cristae formation,^19^ we next assessed cristae organization within RGC axonal mitochondria. The cristae network in *opa1* ^*−/−*^ larval RGC axonal mitochondria is less dense, occupying significantly less of the total mitochondrial matrix (Figure 6P) or is completely devoid of cristae (Figure S9D). Taken together, it is clear that the loss of Opa1 function significantly disrupts mitochondrial organization within zebrafish RGC axons.

While mitochondrial morphology was not extensively examined in other neurons within our *opa1* ^*−/−*^ zebrafish, a preliminary examination of mitochondria within the photoreceptors, which in zebrafish form large structures known as megamitochondria, found evidence of mitochondrial disruption within these structures also (Figure S7L). Specifically, photoreceptor mitochondria were more rounded and the cristae were more globular, with few if any lamellar cristae visible in *opa1* ^*−/−*^ mutants compared to *opa1* ^*+/+*^ siblings. Proteomic analysis reveals no significant changes in the expression of other proteins involved in the regulation of mitochondrial fission or fusion in our loss of *opa1* models (Table S2), suggesting that the morphological changes detected in neuronal mitochondria are due to loss of *opa1*.

**FIGURE 7:**
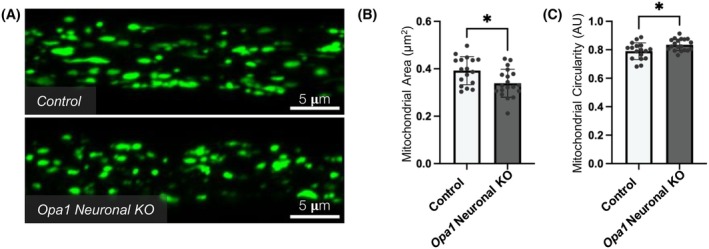
Altered mitochondrial morphology within motor neuron axons in Opa1 neuronal KO *Drosophila*. (A) Representative confocal images of mitochondria (green) within motor neuron axons. Larvae are progeny of *UAS‐Cas9;d42GAL4.UAS‐mitoGFP* flies crossed to *w* ^*1118*^ (control) or *Opa1 sgRNA* (*Opa1* neuronal KO) flies. Graphs represent mean ± SD mitochondrial size (B) and circularity (C). Statistics consist of two‐tailed *t*‐tests.

Within our *Opa1* neuronal KO *Drosophila*, we examined mitochondrial morphology within the axons of long motor neurons. The UAS‐GAL4 expression system enabled us to visualize GFP‐labeled motor neuron mitochondria within axonal bundles (Figure 7A and S10). We found that mitochondria within Opa1 KO motor neuron axons were significantly smaller (Figure 7B) and more circular (Figure 7C) than those in controls. These disruptions in axonal mitochondrial morphology replicate the disruptions identified in *Opa1* ^*−/−*^ zebrafish RGC axons, suggesting that the loss of *Opa1* has a conserved role in regulating mitochondrial organization within neuronal axons in vivo.

### 3.6 Loss of Opa1 compromises mitochondrial function in vivo

**FIGURE 8:**
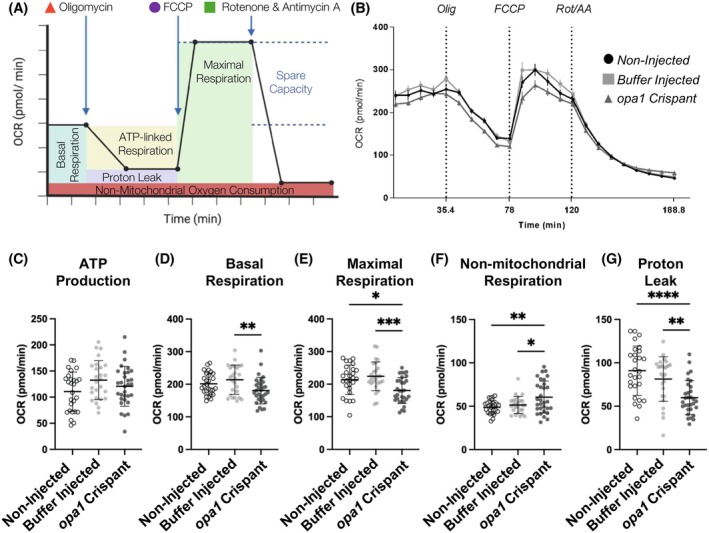
Analysis of mitochondrial respiration in an in vivo model of optic atrophy. (A) Schematic outlining the Mito Stress Test conducted in this study. Oxygen consumption rate (OCR) is measured prior to and following the additions of the ATP synthase inhibitor oligomycin, the uncoupling agent FCCP, and finally the inhibitors of complex I and III, rotenone and antimycin A (respectively). (B) OCR profiles of non‐injected control, buffer injected control, and *Opa1* crispant <131 hpf larvae. (C–G) Graphs represent the mean ± SD OCR for the calculated basal respiration (C), ATP production (D), maximal respiration (E), non‐mitochondrial respiration (F) and proton leak (G). Statistical analysis throughout consists of one‐way ANOVA and Tukey's multiple comparisons tests.

Analysis of both the proteome and axonal mitochondrial organization within our loss of function Opa1 models indicates that mitochondrial function is altered due to loss of *opa1* in vivo. To determine the functional impact of loss of Opa1 on in vivo mitochondrial function, we examined the cellular bioenergetics of *opa1* crispant <131 hpf larvae and buffer injected controls using a Seahorse analyzer to quantify the oxygen consumption rate (OCR) (Figure 8A,B). OCR was measured at regular intervals prior to and following the addition of modulators of respiration (Figure 8C). Basal respiration (Figure 8D) and ATP production (Figure 8E) were not significantly altered in the *opa1* crispant larvae compared to either non‐injected or buffer injected controls. However, *opa1* crispant larvae exhibited significant reduction in their maximal respiration (Figure 8F), a significant increase in their non‐mitochondrial respiration (Figure 8G) and a significant increase in proton leak (Figure 8H) compared to both controls. These findings confirm an essential role for Opa1 in mitochondrial function in vivo.

## 4 DISCUSSION

Autosomal optic atrophy (AOA) is a comparatively common form of inherited retinal disease, causing progressive and irreversible loss of sight associated with the loss of RGCs. While a majority of AOA cases are explained genetically by pathogenic mutations in OPA1, the underlying cause of RGC degeneration in AOA is unknown, and no treatment is presently available. In this paper, we report the creation and characterization of 2 novel in vivo models of OPA1‐mediated AOA, including the first developmentally viable vertebrate *Opa1* KO. These novel models enable us to study in vivo the impact of loss of *Opa*1 in neurons and RGCs specifically, which has previously not been possible. We show that, in addition to a conserved requirement during development, Opa1 has a highly conserved and fundamental role in regulating mitochondrial morphology and function within neuronal axons.

We undertook comprehensive behavioral and phenotypic analyses to understand the consequence of loss of Opa1 on development, survival, plus visual and locomotor function in vivo. Consistent with previous findings, we show that loss of *Opa1* significantly reduces survival. Crucially, however, both neuronal *Opa1* KO in *Drosophila* and homozygous *opa1* KO in zebrafish resulted in viable larvae which can be studied. In *Drosophila*, neuronal *Opa1* KO animals do not survive past 9 days post‐eclosion, with ~50% of flies not surviving beyond 24 h. In zebrafish, homozygous *opa1* KO animals do not survive past 60 dpf (data not shown); the specific reason is unknown. It is not clear why *opa1* KO is better tolerated in zebrafish than in other vertebrate or invertebrate model systems. However, zebrafish models of developmentally lethal mammalian genes have been generated previously and may reflect a differential requirement for the gene product at early cell stages, which is compensated for in zebrafish due to a later transition to zygotic transcription, 512 cells in zebrafish compared to 8 and 1 cells in humans and mice respectively.^46^, ^47^ Of relevance for this study, knockout of Mfn2, which is essential for mammalian development, results in viable, though short‐lived, zebrafish.^46^ Though *Opa1* is reported to be ubiquitously expressed, no gross morphological disruptions were evident in our *Opa1* KO models. In fact, examination of retinal cellular lamination indicates normal retinal cell number at <131 hpf in *opa1* KO zebrafish larvae. Consistent with this, a recent study using RNAi‐mediated knockdown of *Opa1* in *Drosophila* reported that photoreceptor cell bodies were normal at eclosion but subsequently degenerated.^48^ We find that at <131 hpf, visual function is significantly impaired within zebrafish *opa1* KO larva by every test of visual acuity and contrast sensitivity examined.^30^ By contrast, locomotor function is unchanged in these larvae, indicating that retinal neurons are more sensitive to loss of *opa1* than other neurons within zebrafish. Our findings indicate that these novel in vivo models recapitulate key symptomalogical readouts of patients with AOA and provide useful models in which to examine the cellular and molecular underpinnings of neurodegeneration in this disease.

Proteomic profiling of *Drosophila* and zebrafish *Opa1* KO models of AOA provides a novel insight into the molecular pathways altered by loss of *Opa1* in both models. Apart from the product of *Opa1* itself, which, as expected, was significantly decreased in both models, very little overlap in individual differentially expressed (DE) proteins was detected. Within *Opa1* neuronal KO *Drosophila*, proteins that function to regulate synaptic organization and signaling were particularly altered, suggestive of neuronal dysfunction. By contrast, within *opa1*‐deficient zebrafish, proteins that function in translation pathways were highly reduced, suggesting a decrease in global protein synthesis, but not large‐scale neurodegeneration. This difference likely reflects the more advanced neurodegenerative disease state in 1‐day post‐eclosion *Opa1* neuronal KO *Drosophila* compared to <131 hpf *opa1* KO zebrafish, in which no neuronal loss is detected. Notwithstanding these differences, a striking finding of the proteomic analysis was that both *Opa1* KO models resulted in a highly conserved enrichment of proteins associated with the IPA category “*mitochondrial dysfunction*” with marked decreased expression of constituents of the respiratory complexes I and III. Evidence to date has been inconclusive as to whether *Opa1* mutations affect the function of respiratory complexes, with some in vitro models showing loss of expression, whereas others showing no change.^22^, ^49^ Our findings support a model whereby respiratory complexes I and III may be particularly sensitive to *Opa1*‐associated defects within neurons in vivo. Interestingly, proteins known to regulate mitophagy or mitochondrial biogenesis were not significantly dysregulated in our loss of *Opa1* models. This does not preclude the involvement of these pathways within our models; however, it suggests that these pathways are not highly disrupted at time points where we detect significant behavioral and molecular changes.

Within long neuronal axons, i.e. motor neuron axons in *Opa1* neuronal KO *Drosophila* and RGC axons in *opa1* KO zebrafish, *Opa1* disruption results in fragmentation of the mitochondrial network and accumulation of mitochondria with disordered cristae organization. This is consistent with the increased mitochondrial fragmentation seen in many previous in vitro and in vivo models of *Opa1* deficiency, and in mitochondrial fusion disorders more broadly.^20^, ^48^, ^50^, ^51^ Interestingly, our analysis of mitochondrial respiratory function reveals that, despite these marked morphological defects, mitochondria within *opa1* deficient <131 hpf zebrafish are still able to meet the energetic needs of the larvae, i.e. overall ATP production is unchanged. However, a significant decrease in the maximal respiration suggests that mitochondria in *opa1* deficient zebrafish cannot work to the same capacity, providing direct evidence that *Opa1* is required for normal mitochondrial respiration in vivo. Importantly, our findings demonstrate that *Opa1‐*induced RGC axonal mitochondrial fragmentation and visual impairment precede loss of RGCs.

In conclusion, we have generated two novel in vivo models of *Opa1*‐associated AOA. Both models replicate features previously observed within patient‐derived samples, including disrupted mitochondrial proteins and a fragmented mitochondrial network within long axons. Importantly, zebrafish *opa1* deficient models replicate the loss of visual function experienced by patients. These models provide a unique opportunity to rapidly screen therapeutics for their ability to rescue vision, a highly relevant patient outcome, in a well‐established model system.^52^, ^53^ Given the decreased expression of respiratory complexes I and III and the impaired mitochondrial respiratory function identified in our *Opa1* models, it might be predicted that agents which promote respiratory function would lessen the effect of *Opa1* disruption in affected neurons. Indeed, idebenone, the coenzyme Q10 analogue that transfers electrons from complexes I and II to complex III, has shown some promise in pilot studies for AOA and other mitochondrial diseases.^54^, ^55^ However, in our hands, treatment with idebenone did not significantly improve survival, visual function, or mitochondrial organization in our *Opa1* models (data not shown). While this may imply that it is not sufficient to rescue a potential respiratory defect at this point in the electron transport chain in this model system, our findings do support the hypothesis that therapeutics which support the function of respiratory chain complexes may prove efficacious for the treatment of AOA.

## AUTHOR CONTRIBUTIONS

NCO'S and BNK contributed to the study conception and design. Material preparation, data collection and analysis were performed by ELS. The first draft of the manuscript was written by ELS, BNK and NCO'S. BD contributed to the conception and design of the Seahorse experiment. AP, KD, and JR realized, collected, and analyzed the Seahorse experiment. MS and JCG aided with the conduction of Ingenuity Pathway Analysis and advice on results interpretation. ETD contributed advice and assistance on proteomics sample preparation, carried out the mass spectrometric analysis, searched the raw data, and performed hierarchical clustering analysis.

## FUNDING INFORMATION

ELS was supported by an IRC‐Government of Ireland Ph.D. studentship (Grant number GOIPG/2020/1312). This work in BNK's laboratory was supported by a research grant from Science Foundation Ireland (SFI) (Grant number 20/FFP‐P/8538). NCO'S was supported by a UCD Ph.D. Advance award. Work in the BD lab is funded by grants from external resources of the University of Montpellier; Agence Nationale pour la Recherche [ANR‐23‐CE14‐0087]; AFM (24197).

## DISCLOSURES

The authors declare that the research was conducted in the absence of any commercial or financial relationships that could be construed as a potential conflict of interest.

## Supporting information


**Data S1:** Supporting Information.

Video S1:

## Data Availability

Access to the raw data supporting the findings of this study on ProteomeXchange are available from the corresponding author on request.
