# Supplementary material for: Novel in vivo models of autosomal optic atrophy reveal conserved pathological changes in neuronal mitochondrial structure and function
Source: FASEB J. 2025 Apr 9;39(7):e70497. doi: 10.1096/fj.202403271R (PMC11981028; doi:10.1096/fj.202403271R)
Supplement: Supplementary file 1 — Data S1: Supporting Information. [file FSB2-39-e70497-s001.pdf]

Supplementary Figures

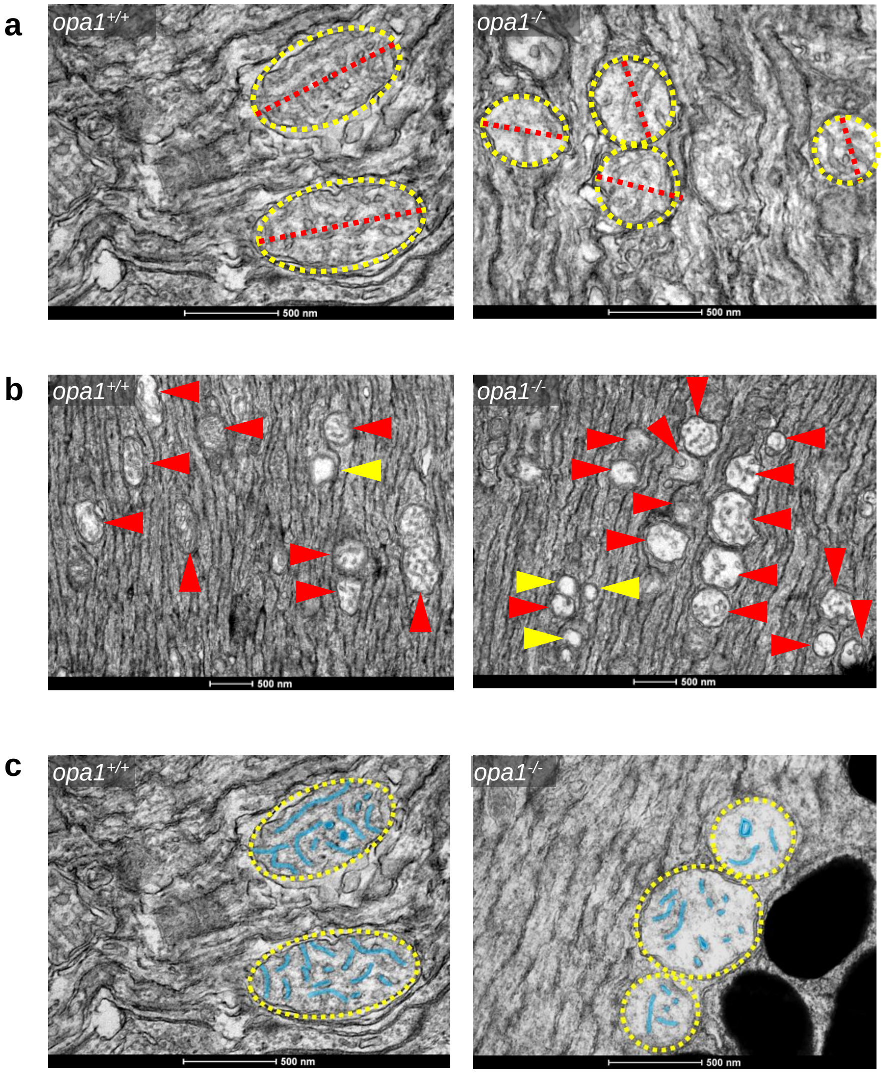

Supplementary Figure 1. Quantification of mitochondrial morphology in electron microscopic images of zebrafish optic nerve. Shown are representative electron microscopic images of mitochondria within zebrafish optic nerves. (a) Shows bounding area of individual mitochondria (yellow dashed line) which were used to define: length of longest mitochondrial axis (red dashed line); mitochondrial circularity and mitochondrial area in ImageJ. (b) Individual mitochondria are indicated with red arrowheads. Examples of mitochondria devoid of any internal structure are indicated with yellow arrowheads. (c) Shows individual cristae within a mitochondrion (blue). The total area of cristae was calculated and expressed as a percentage of the total mitochondrial area (yellow dashed line).

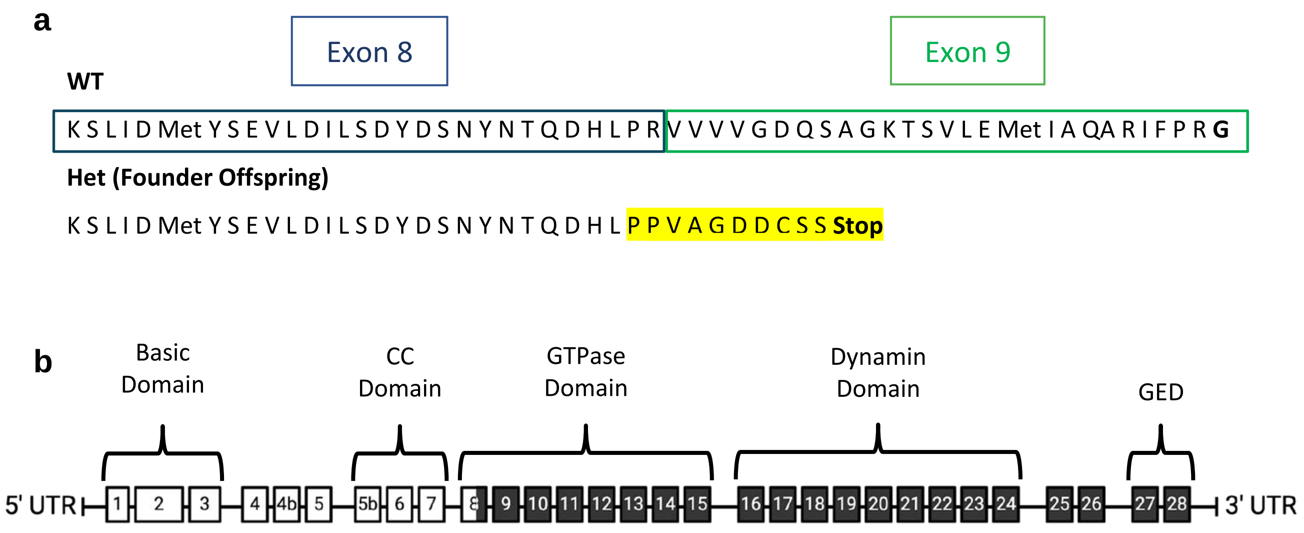

Supplementary Figure 2. Details of the germline Opa1 deletion created in this study. (a) aa sequence of exons 8 and 9 of WT zebrafish Opa1 (top) compared to the predicted sequence of exons 8 and 9 in the founder animal of the *opa1*$^{+/-}$ stable line (bottom). (b) Schematic of the WT *opa1* gene with the exons predicted to be lost with the premature STOP codon indicated in black. cc domain = coiled coil domain; GED = GTPase effector domain.

```
1                                                                               82
TTGAAGTACCAACGGATGCTTGAGCGCTTGGAAAAGGAAAATAAAGAATTGAGGAAAGTTGTATTGCAGAAGGATGACAAGG
T-----TACCAACGGATGCTTGAGCGCTTGGAAAAGGAAAATAAAGAATTGAGGAAAATTGTATTGCAGAAGGATGACAAGG
..................................................................................

83                                                                               164
GTATTCACCAAAGAAAAGTCAAGGTCAGATTTCAGTTGTTTTCTTTCAGATTGAAAATGTTTTCACATTTTACAGATGAAA
GTATTCACCAAAGAAAAGTCAAGGTCAGATTTCAGTT---TTTCTTTCCGATTGAAAATGTTTTCATATTTTACAGATGAAA
..................................................................................

165                                                                              246
ACAAAACAGATCACTGGGTTATATTAATGCATTTCTCCTATTGGTCTCGTGCAGAAATCTTTGATTGATATGTACTCTGAGG
ACAAAACAGATCACTGGGTTATATTAATGCATTTCTCCTATTGGTCTCGTGCAGAAGTCTTTGATTGATATGTACTCTGAGG
..................................................................................

247                                                                              328
TCCTGGACATTCTTTCAGATTATGACTCCAACTACAACACTCAAGATCACCTGCCAAGGGTTAGTCTTATTCCTTTTTTTC
TCCTGGACATTCTTTCAGATTATGACTCCAACTACAACACTCAAGATCACCTGCCTCCAGTTGCT-----------------
..................................................................................

329                                                                              410
ATTATTTGAAATATTTGCTTAAAGCTTTTCATTACCTTTTCAAATAGAAATGTTTTTGTTTTCAAAAATGCCAATACAATAT
----------------------------------------------------------------------------------
..................................................................................

411                                                                              492
ATGCTTAAGTGAATTGAGATGGTTCAATGTACTGTAAATGCAAGCACACACACCGTTTTTAGATTTTTAAAACTCTTTTCAG
----------------------------------------------------------------------------------
..................................................................................

493                                                                              574
GATTAACCAATGAGACACAATAGCTGTGTCTCATTTCAGAGGCTGCATTCTCCAAAGGATGCATGTGAAGGGTGCTACGTCA
----------------------------------------------------------------------------------
..................................................................................

575                                                                              656
TCGTGGTGCAACGAAGGCTGTCTTTTTAGAAAAATATTCAAAGGCTCCTTCAAATGTAGCCTCCAAATGTGTCCTTTGTTTC
----------------------------------------------------------------------------------
..................................................................................

657                                                                              738
CCAATTAATGAAGGACTTAACCAGTGGATCCTTTGCGGCCTCAATCATCCTAGGGTTCATTGTGTGCTGATAACATTTTTAA
----------------------------------------------------------------------------------
..................................................................................

739                                                                              820
AGGAAACTCTGGATTAAATGTAGGAATTATCTTTATTCCTGCCAAGGGTTAGTCTTATTCTTACTTAAATATCTACACACAT
----------------------------------------------------------------------------------
..................................................................................

821                                                                              902
GTTGCAGAGCATGACATGTCTTACTAAAAAAAGATTTTATAGACAGTTTTATGTGTACATGTATGGATTTACTGTAAGGAAT
----------------------------------------------------------------------------------
```

```
903                                                                            984
TATATTTCCGGTTTCTGTAAACCTTGCTTTATTTTGATGCCAAAAACCTGGAATCATTGACTTCCATAATATTTGCTTTTCC
----------------------------------------------------------------------------------

..................................................................................

985                                                                           1066
TACTATAAAATTTAGGGGTTATGGGTTTTCAGCTTTCTTCAAAATGGCTTTTTCAGTGTTCAACAGAATAAACAAGCTCATT
----------------------------------------------------------------------------------

..................................................................................

1067                                                                          1148
AAAGGTTTGAAACCACTTGAGGGAGGATAAATAATGTGTAAATTGTTATTAGAACTTTATTATTATTACTACTATCCAGTTA
----------------------------------------------------------------------------------

..................................................................................

1149                                                                          1230
ATGGATCAGACATAAACAGATTCTGTCTCCTCTTTTTTTAGGTGGTGGTAGTTGGGGACCAGAGTGCGGGAAAGACTAGTGT
----------------------------------------------------------------------------------

..................................................................................

1231                                                                          1312
GCTGGAGATGATTGCTCAAGCTAGGATCTTTCCGAGAGGCTCTGGAGAAATGATGACAAGGTCTCCTGTCAAGGTGAACATG
---GGAGATGATTGCTCAAGCTAGGNTCTTCCGAGNNNNNNNNNNNNNNNNNNNN---------------------------

..................................................................................

1313                                                                          1394
CTGTTTTTTTTTGCTGTTTGTGTTGGAAACCTGAAATACGTCACCTATGTGATTTGTGGAGTTATTTTTCAGAATGATGGCT
----------------------------------------------------------------------------------

..................................................................................

1395                                              1446
GATAGTATTTATGTACAGTGATTATTTAAACTGTAATGTTGCTTAAGGGCAC
----------------------------------------------------
```

Supplementary Figure 3. Mutant zebrafish Opa1 genomic sequence alignment. Sequence alignment of WT zebrafish Opa1 exons 8 and 9 compared to the sequence of exons 8 and 9 in the stable mutant allele.

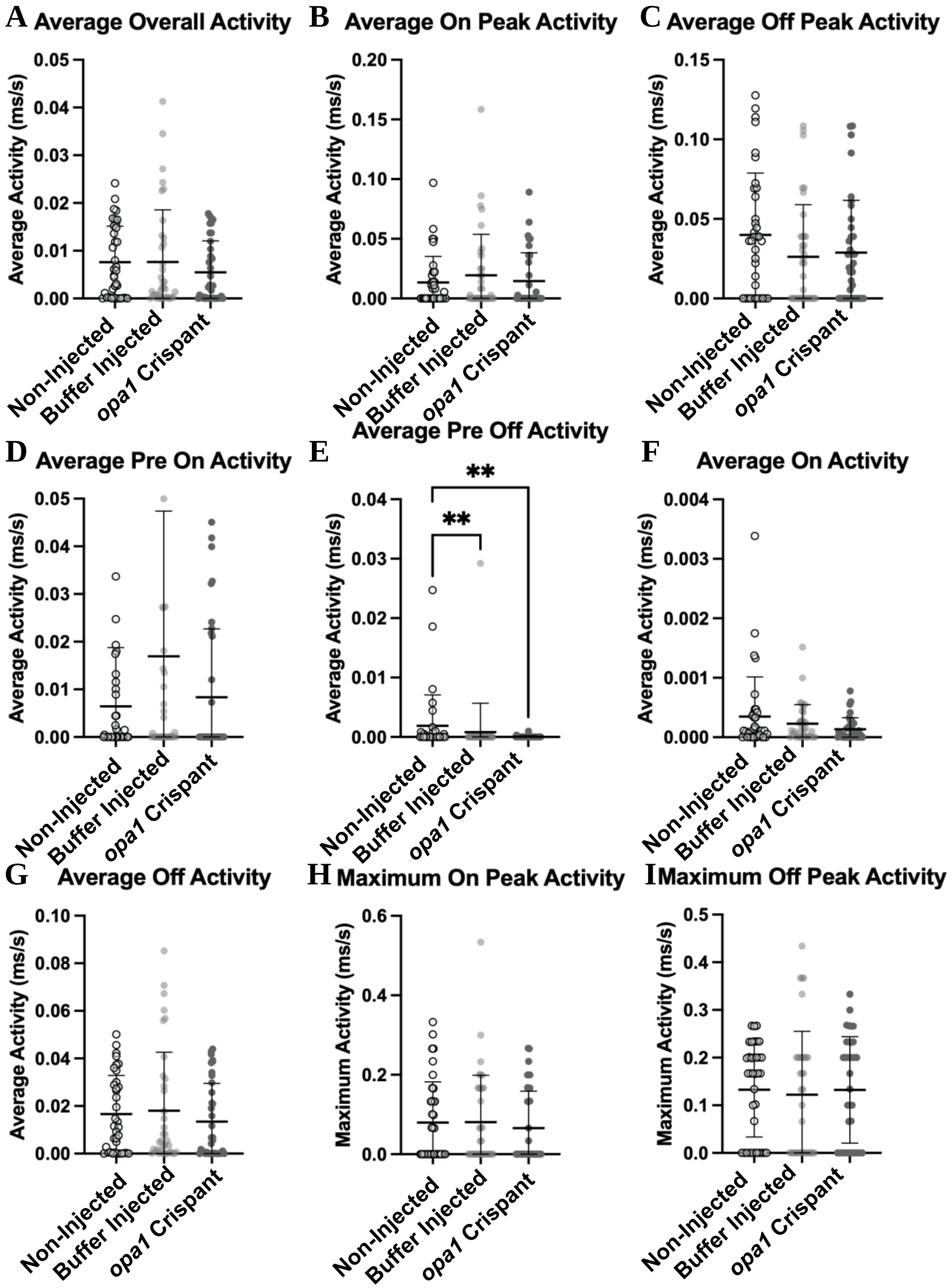

Supplementary Figure 4 Crispant zebrafish Larvae VMR Analysis. N=3, n=12 for each group. (A) Overall activity includes the entire recording period (B,C) On and off peaks include the activity for 5 seconds after the lights turn on or off respectively. (D,E) Pre on and off periods represent the 100 seconds prior to the lights turning on or off respectively. Crispants and buffer injected animals have significantly ($p<0.01$) reduced activity compared to non-injected siblings. (F,G) Average on and off represents the mean activity for the lights on or off periods. (H,I) Maximum on or off peaks represent the maximum activity value for each animal for the 5 seconds following the lights turning on or off.

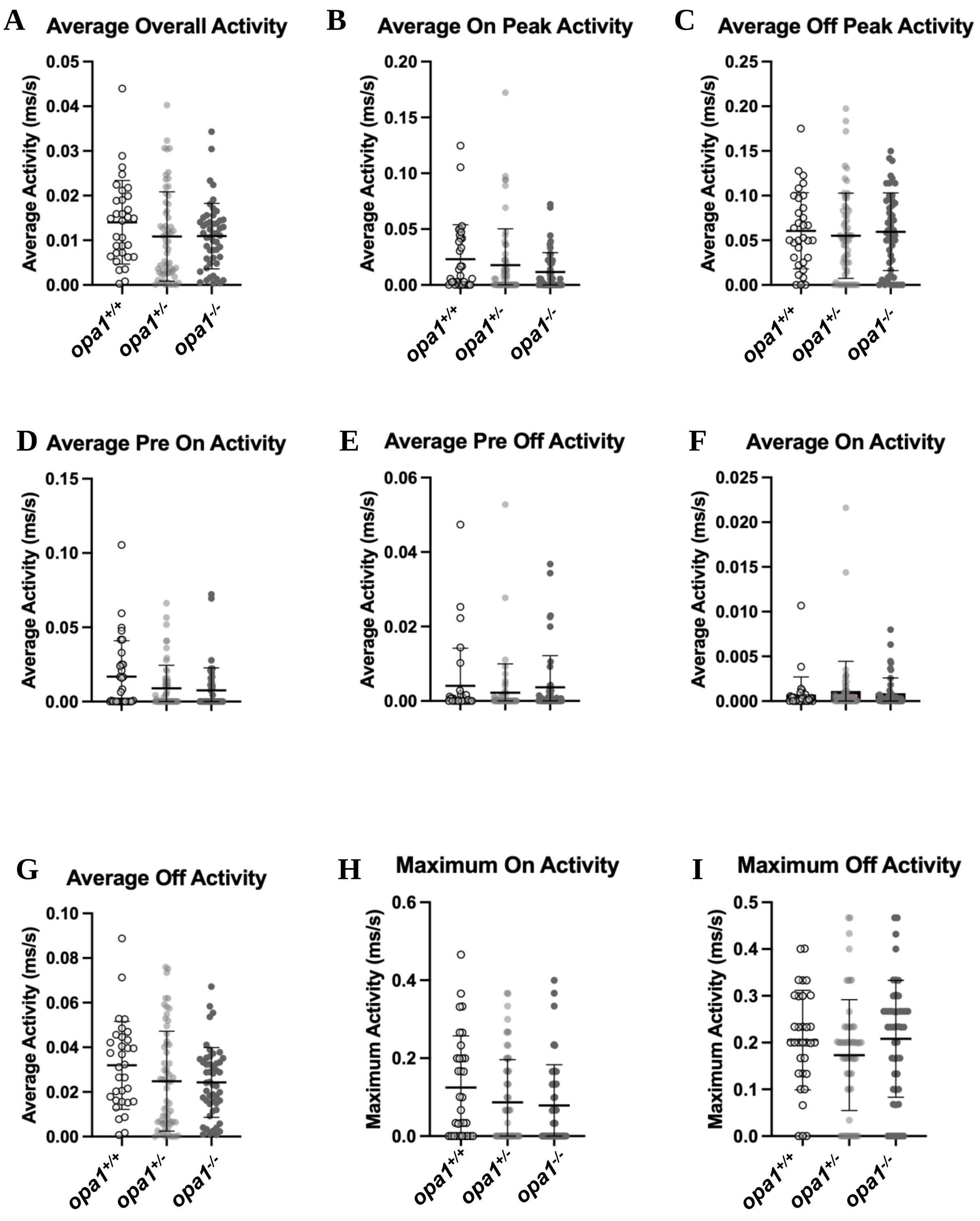

Supplementary Figure 5. Stable zebrafish Opa1 KO VMR Analysis: (A-I) Average larval activity across all time periods for VMR assays. No significant differences were found between *opa1*$^{-/-}$ larvae and their WT or heterozygous siblings during any period. N=3, n=48 total animals per replicate prior to genotyping.

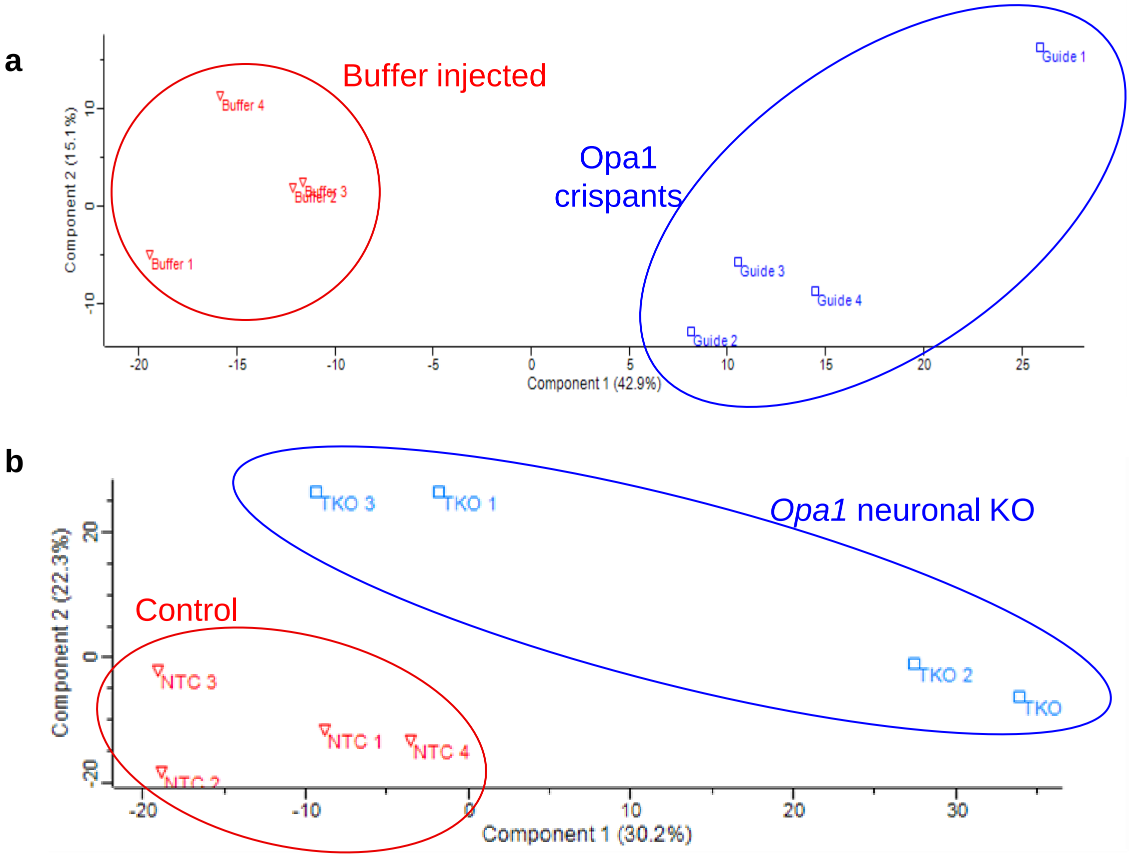

Supplementary Figure 6. Principal component analysis (PCA) of proteomic datasets generated this study. Variation analysis of samples used in proteomic analysis. PCA analysis of (a) zebrafish and (b) *Drosophila* Opa1 models and controls from the proteomic dataset, generated using Perseus software.

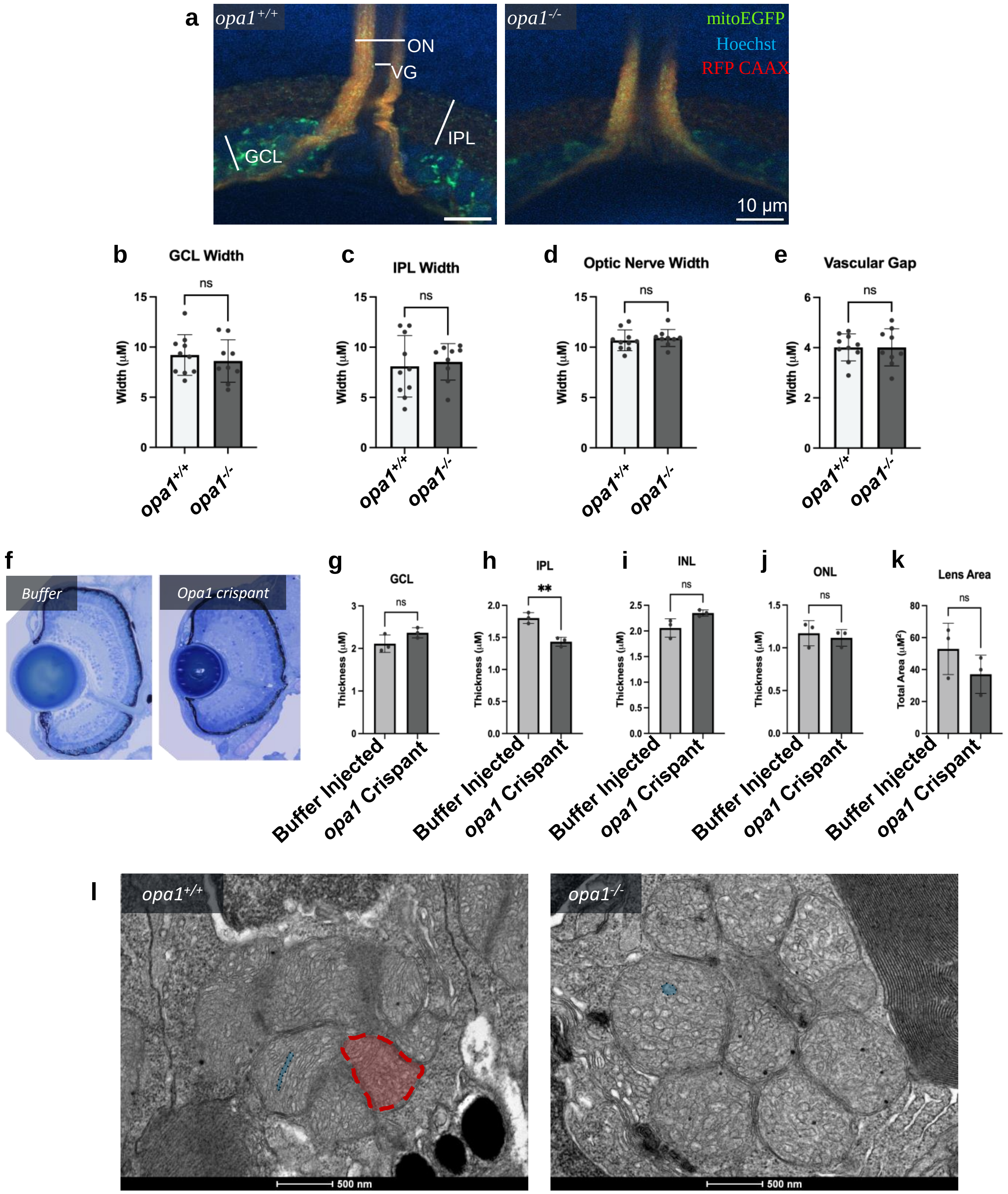

Supplementary Figure 7. Additional zebrafish imaging Data: (a) Representative Airyscan deconvoluted images of 5dpf retinas of *opa1*$^{+/+}$ and *opa1*$^{-/-}$ larvae in a *Tg(isl2b:mitoeGFP-2aTagRFPCAAX)* background. (b-e) Measurement of the width of (b) GCL, (c) IPL, (d) ON or (e) the space within the optic nerve through which the vasculature passes did not show any differences between *opa1*$^{+/+}$ and *opa1*$^{-/-}$ larvae (N=9-10). (f) Representative toluidine blue stained sections of 5 dpf zebrafish retinas, of buffer injected and *opa1* crispant larvae. (g-k) Average widths of 5dpf buffer vs. crispant retinal layers, shown as mean ± SD thickness in µm of the (g) GCL (h) IPL (i) INL or (j) ONL respectively. (N=3) (k) is the total area of the lens in µm^2 (N=3). (l) TEM images of megamitochondria within the photoreceptors of *opa1*$^{+/+}$ and *opa1*$^{-/-}$ <131 hpf larvae.

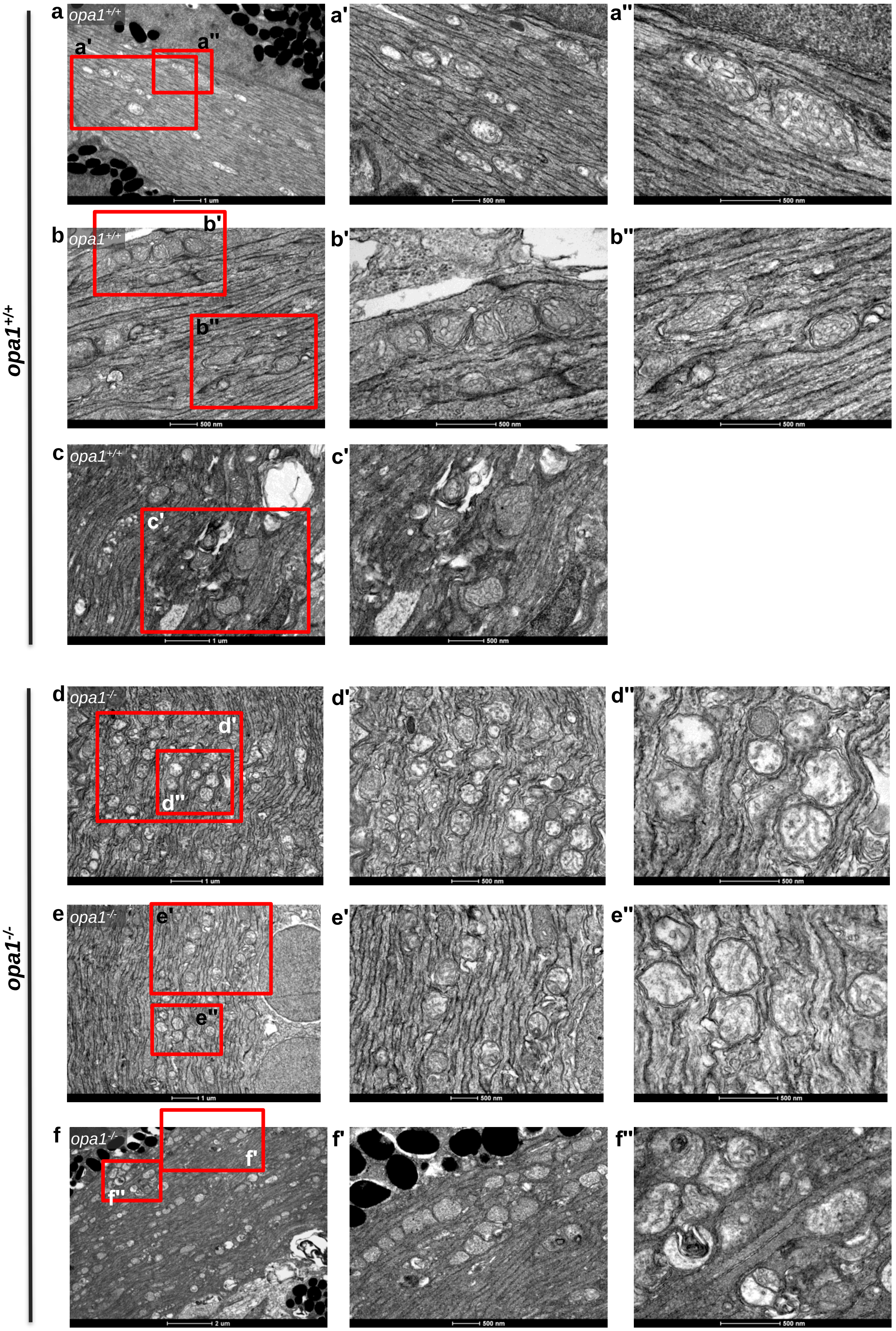

opa1+/+
opa1-/-
a
opa1+/+
a'
a''
1 um
500 nm
b
opa1+/+
b'
b''
c
opa1+/+
c'
d
opa1-/-
d'
d''
e
opa1-/-
e'
e''
f
opa1-/-
f'
f''
2 um

Supplementary Figure 8. Electron microscopic analysis of the optic nerve within opa1$^{-/-}$ zebrafish and WT siblings. Shown are representative electron microscopic images of optic nerves from 3 independent zebrafish per genotype: *opa1*$^{+/+}$ 1-3 (a-c respectively) and *opa1*$^{-/-}$ 1-3 (d-f respectively).

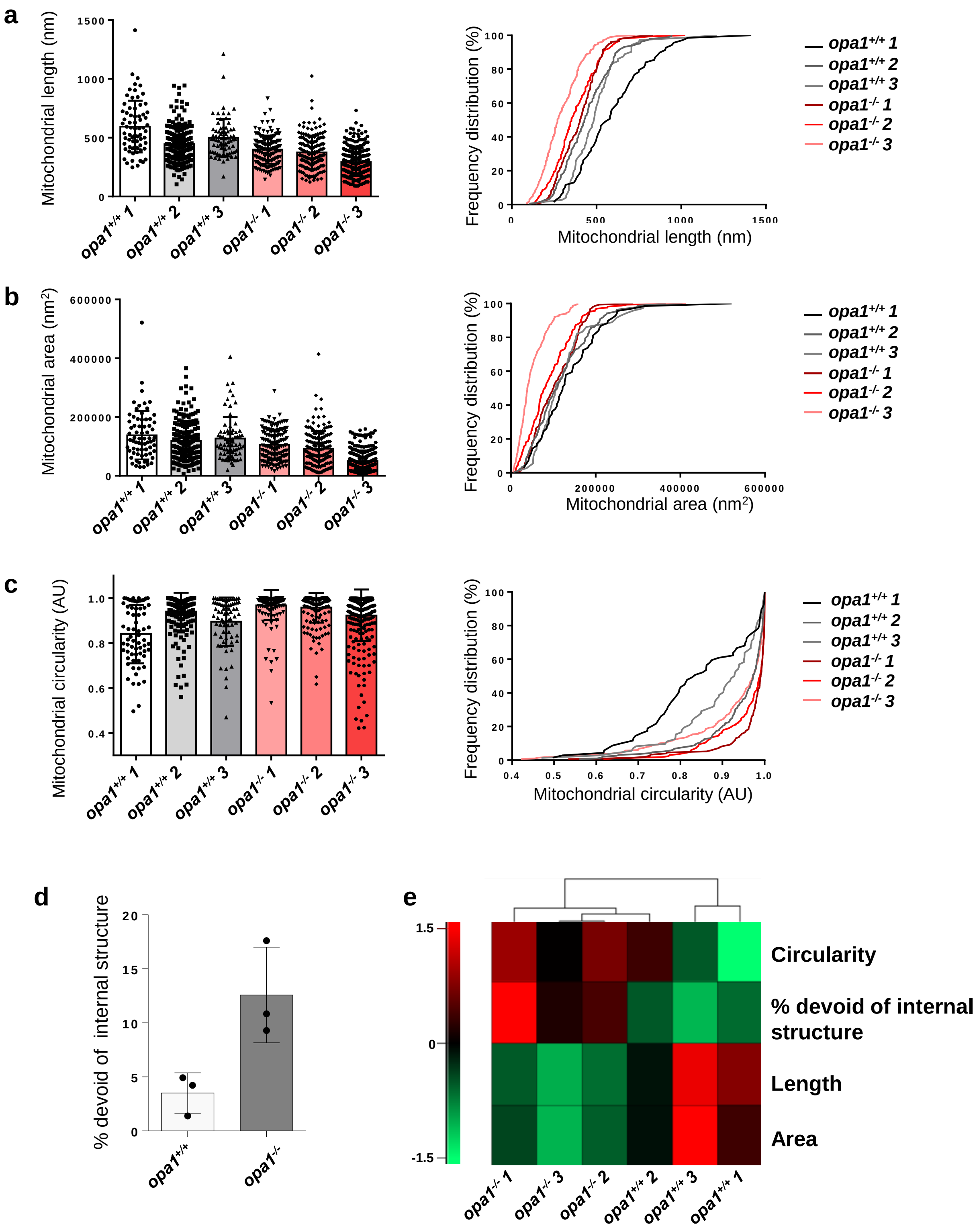

Supplementary Figure 9. Comparison of morphological metrics between mitochondria within zebrafish optic nerve. Mitochondrial length (a), area (b) and circularity (c) with their respective cumulative frequency distribution for each *opa1*$^{-/-}$ and *opa1*$^{+/+}$ sibling. n = 69 – 280 mitochondria per larval optic nerve. (d) Shown are % mitochondria devoid of internal structure for each optic nerve. n = 3. (e) Heatmap across larval average for each mitochondrial morphological metric measured with dendrogram illustrating hierarchical clustering of pattern similarity across morphological parameters and samples (Euclidean distance measure, k-means clustering algorithm). The colours indicate the relative quantitative value, where red indicates a higher value, and green indicates a lower value.

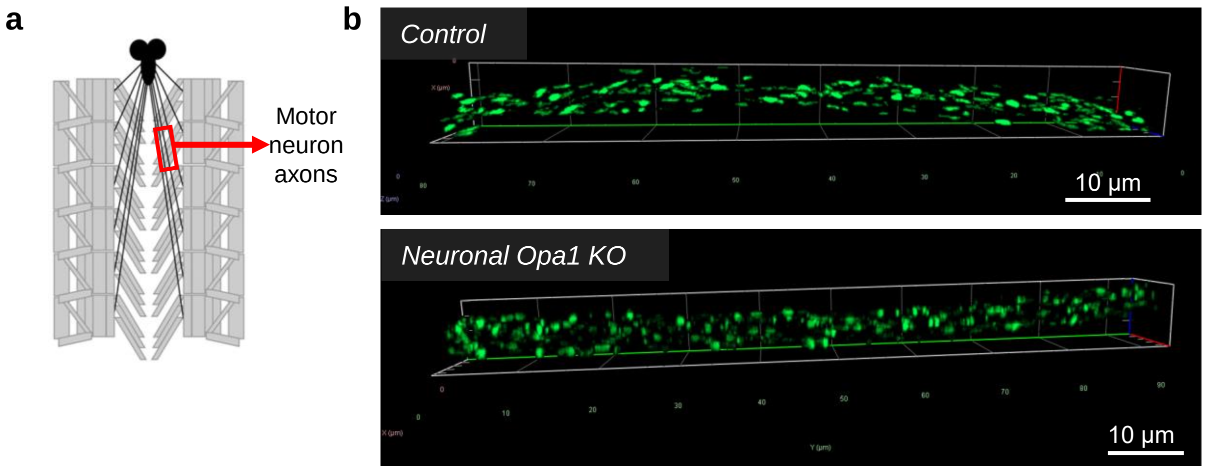

Supplementary Figure 10. Mitochondrial morphology in neuron-specific Opa1 KO *Drosophila* axons. (a) Schematic diagram illustrates the organisation of long motor neuron axons in *Drosophila* larvae which were imaged in this study. (b) 3-dimensional reconstructions of mitochondria within motor neuron axons generated by maximum projection of Airyscan z-stack images. Images are acquired from the progeny of *UAS-Cas9;d42GAL4.UAS-mitoGFP* flies crossed to w^{1118} (control) or *Opa1 sgRNA* (Opa1 neuronal KO) flies.

Supplementary Tables

Supplementary Table 1. List of primers used for PCR and qPCR analyses in this study.

Zebrafish Primers (PCR and qPCR)	
Primer Name	Sequence 5’-3’
Opa1 Forward	TACCAACGGATGCTTGAGCG
Opa1 Poison	GGGAAACAAAGGACACATTTGGA
Opa1 Reverse	AACAGCATGTTCACCTTGACAG
Opa1 Forward (qPCR)	AAAGACTAGTGTGCTGGAGATG
Opa1 Reverse (qPCR)	GACAGGAGACCTTGTCATCATT
β-actin Forward (qPCR)	CTTCCTGGGTATGGAATCTTGC
β-actin Reverse (qPCR)	GTGGAAGGAGCAAGAGAGGTG
Drosophila Primers (qPCR)	
Primer Name	Sequence 5’-3’
Opa1 Forward	TGCACAGTCAGGTTCTCAAAA
Opa1 Reverse	TATGAATTCCTGCTGCAACG
Rp49 Forward	CGGATCGATATGCTAAGCTGT
Rp49 Reverse	CGACGCACTCTGTTGTCG

Supplementary Table 2. Summary of expression levels of mitochondrial fission/fusion regulating proteins as determined by proteomic analysis. Values not reproducibly detected are indicated as ND.

Human Gene Ortholog (Uniprot ID)	Zebrafish			Fruit Fly		
	Ortholog (Uniprot ID)	Buffer Injected LFQ Value [log2]	*opa1* Crispant LFQ Value [log2]	Ortholog (Uniprot ID)	Control LFQ Value [log2	*Opa1* Neuronal KO LFQ Value [log2
MFN2 (O95140)	Mfn2 (A0A8M2BJP0)	ND	ND	Marf (Q7YU24)	0.61	-0.61
MFN1 (Q8IWA4)	Mfn1a (A0A8M6YSN1)<br>Mfn1b (Q6PFP9)	ND<br>ND	ND<br>ND	Fzo (O18412)	ND	ND
OMA1 (Q96E52)	Oma1 (E9QBI7)	ND	ND	Ortholog Unclear	ND	ND
YME1L (Q96TA2)	Yme1l1a (E7EZJ5)<br>Yme1l1b (A0A0R4IJC7)	ND<br>ND	ND<br>ND	Yme1l (F3YDF1)	ND	ND
DNM1L/DRP1 (O00429)	Dnm1l (Q7SXN5)	28.16	28.05	Drp1 (Q9VQE0)	0.55	-0.55
FIS1 (Q8IWA4	Fis1 (A0A2R8Q8G0)	ND	ND	Fis1 (B7YZT2)	ND	ND
